# Trends in Measures of Child and Adolescent Well-being in the US From 2000 to 2019

**DOI:** 10.1001/jamanetworkopen.2022.38582

**Published:** 2022-10-26

**Authors:** Nathaniel W. Anderson, Daniel Eisenberg, Neal Halfon, Anna Markowitz, Kristin Anderson Moore, Frederick J. Zimmerman

**Affiliations:** 1 Department of Health Policy and Management, UCLA (University of California, Los Angeles); 2 Department of Public Policy, UCLA; 3 Department of Pediatrics, UCLA; 4 Department of Education, UCLA; 5 Child Trends, Bethesda, Maryland

## Abstract

**Importance:**

Improving child and adolescent well-being is a critical public health goal, yet monitoring of this measure at the national level remains limited. Composite indices aggregate existing indicators from population data sources, but these indices currently have weaknesses that may inhibit widespread use.

**Objective:**

To apply a novel, more child-centric index method to document changes in overall child and adolescent well-being in the US from 2000 to 2019, assess which states and racial and ethnic subpopulations experienced the greatest inequities in well-being, and identify the specific components associated with changes in the index over time.

**Design, Setting, and Participants:**

This cross-sectional study applied the Child and Adolescent Thriving Index 1.0 to population-level data from 2000 to 2019 from several data sources. The area-based sampling frame for each of the component data sources allowed for nationally representative estimates for every year of the study period. The indices for every state and by race and ethnicity were also calculated. Due to the scope and breadth of the index components from across the life course, the Child and Adolescent Thriving Index 1.0 is intended to approximate the well-being of persons up to age 17 years. Data were analyzed from June 7, 2021, to March 17, 2022.

**Exposures:**

Time in years.

**Main Outcomes and Measures:**

The Child and Adolescent Thriving Index 1.0 is a weighted mean of 11 indicators intended to proxy well-being. The index comprises 11 components: non–low birth weight in neonates, preschool attendance in children aged 3 to 4 years, reading proficiency in fourth-grade students, math proficiency in eighth-grade students, food security in children younger than 18 years, general health status, nonobesity in high school students, nonsmoking in adolescents aged 12 to 17 years, non–marijuana use in adolescents aged 12 to 17 years, high school graduation in young adults aged 18 to 21 years, and nonarrest rate in children aged 10 to 17 years. The index ranges from 0 to 1, with 0 indicating minimum and 1 indicating maximum possible well-being at the population level.

**Results:**

The Child and Adolescent Thriving Index 1.0 was applied to data from 12 320 national, state, and racial and ethnic population-level estimates. Over the study period, the Child and Adolescent Thriving Index 1.0 score increased from 0.780 points in 2000 to 0.843 points in 2019. Despite some convergence in geographic and racial and ethnic disparities, inequities were still present in 2019 in the South (−0.021 points) compared with the Northeast and among American Indian or Alaska Native (−0.079 points), Black (−0.053 points), and Latinx (−0.047 points) children and adolescents compared with White youths. Index components most associated with the overall increases in index scores of well-being were high school graduation rate (+0.028 units) and nonsmoking in adolescents (+0.022 units), amounting to 80.6% of the total increase.

**Conclusions and Relevance:**

Results of this study suggest that child and adolescent well-being scores increased from 2000 to 2019, but substantial work remains to address persistent inequities across states and racial and ethnic populations. The newly developed Child and Adolescent Thriving Index 1.0 may be used in future work to evaluate which public policy types (economic, social, health care, housing, or education) are associated with higher levels of well-being.

## Key Points

**Question:**

What were the trends and existing disparities in child and adolescent well-being in the US between 2000 and 2019?

**Findings:**

In this cross-sectional study that applied a novel composite index, the Child and Adolescent Thriving Index 1.0, to data from 12 320 national, state, and racial and ethnic population-level estimates spanning a multidimensional set of population data indicators that proxy for child and adolescent well-being. Results show national well-being index scores increased steadily from 2000 to 2019, but disparities by geographic region and race and ethnicity persisted.

**Meaning:**

Findings of this study suggest that, despite increases in index scores measuring well-being over the past 2 decades, substantial work remains to be done to address the enduring inequities in child and adolescent well-being.

## Teaser

This cross-sectional study describes the Child and Adolescent Thriving Index 1.0, a new well-being measurement method that uses population-level data.

## Introduction

There is a general sense among many observers that children and adolescents in the US are not thriving. Compared with their peers in other advanced nations, US youths experience more unfavorable outcomes across numerous health and social indicators, including mortality, family poverty, access to health care, and exposure to crime.^1,2^ Of particular concern are well-documented inequities by race and ethnicity and socioeconomic class.^3,4,5^

Many governments across the world have developed expansive data and monitoring systems with the goal of tracking younger peoples’ wide-ranging and multidimensional needs.^6,7^ However, the extensive set of available indicators may obscure how children and adolescents are faring overall. For example, measures in the US, such as teen birth, infant mortality, and poverty rates, improved from 2018 to 2019, whereas other measures, such as preterm birth and adolescent depression rates, worsened.^8^ With little guidance on which subsets of these indicators are most essential to young peoples’ quality of life, there remain open questions on whether progress is being made in overall well-being.

Given that a single summary indicator might reasonably describe the overall status of children and adolescents,^9^ researchers have developed numerous indices of well-being.^10^ However, these indicators lack focus on the actual lived experiences of younger people.^11^ Specifically, they overlook subjective well-being entirely; conflate contextual measures with more direct individual outcomes; and assume equally weighted components, which may inaccurately represent their actual contribution to well-being.

The aims of this study were as follows: (1) to apply a novel, more child-centric index method to document changes in overall child and adolescent well-being in the US from 2000 to 2019; (2) to assess which states and racial and ethnic subpopulations experienced the greatest inequities in well-being; and (3) to identify the specific components associated with changes in the index over time.

## Methods

This repeated cross-sectional study applied the Child and Adolescent Thriving Index 1.0, a weighted mean of 11 indicators intended to proxy the well-being of persons up to age 17 years, for the period from 2000 to 2019 in the US. The University of California, Los Angeles Institutional Review Board deemed this study exempt from review and waived the informed consent requirement due to the use of deidentified data. We followed the Strengthening the Reporting of Observational Studies in Epidemiology (STROBE) reporting guideline.^12^

### Literature Review

Well-being has a number of definitions spanning many fields.^6^ We prefer this definition from the Robert Wood Johnson Foundation: “the comprehensive view of how individuals and communities experience and evaluate their lives, including their physical and mental health and having the skills and opportunities to construct meaningful futures.”^13^^(p7)^

Child and adolescent well-being indices measure this construct at the population level by compiling data across numerous large-scale sources to cover the various aspects affecting younger peoples’ lives and then aggregating these components into a single measure. In the US, the most prominent example is the Annie E. Casey Foundation’s KIDS COUNT index, which has annually ranked states’ levels of well-being since 1990.^14^

One of the key strengths of such indices is that the underlying data are reliably collected over time, allowing for analysis of trends. According to the KIDS COUNT index and its previous iteration,^15^ well-being has improved over the past 4 decades.^14,15,16,17^ In state rankings, those in the Northeast and Upper Midwest tend to perform above average, whereas states in the Sunbelt fare worse. There are large inequities by race and ethnicity and household income, although there has been some convergence during periods of economic growth.^18^ Decomposition analysis suggested that overall advances from 2000 to 2015 were largely attributable to improvements in risky behaviors and community engagement, whereas measures of physical health and emotional well-being declined.^15^

However, these indices have certain limitations that may inhibit widespread use.^17,18,19^ First, the components of these indices are not grounded in younger peoples’ subjective experiences,^7^ which is a critical omission because subjective and objective perspectives have been shown to be related but distinct constructs that each contribute to understanding well-being.^20,21^ Second, components may not be truly child centered. Rather than directly measure outcomes at only the individual level, indices adopt components of the broader context that shape well-being.^22,23^ However, if the aim of a policy is to improve well-being by shaping the context, then the outcome measure should recognize that context is a mediator of well-being and therefore distinct from it. Counting the mediating context as part of the outcome could lead to a false impression of the effectiveness of improving that context by, in a sense, double-counting. Third, index components are typically weighted equally, which may not reflect the components’ actual respective associations with overall well-being.^11,24^

We applied an index that addresses these weaknesses: the Child and Adolescent Thriving Index 1.0. Briefly, we used data from a nationally representative longitudinal panel to identify index components and their respective weights. Rather than include measures of mental health or subjective well-being as several components among many others, we regressed these measures against a set of individual-level index components. These components were restricted such that they excluded purely contextual measures that can be objectively observed, such as poverty and parental unemployment, while retaining indicators of how individuals subjectively experience their surrounding context, such as food insecurity. The resulting index more accurately proxies the latent construct of well-being while still relying on indicators already collected in population-level data sources. More information about the Child and Adolescent Thriving Index 1.0 can be found in previously published work.^25^ Within the panel, the index shows strong predictive validity with future health and economic outcomes and performs similarly to a version of the KIDS COUNT index. However, up to this point, the Child and Adolescent Thriving Index 1.0 has not been evaluated nationally using population-level macrodata.

### Data Sources and Index Construction

For this study, we collected national and state-level macrodata on index components from a variety of sources, including the American Community Survey; Centers for Disease Control and Prevention National Center for Health Statistics, National Vital Statistics Reports; Current Population Survey; Office of Juvenile Justice and Delinquency Prevention Statistical Briefing Book; Substance Abuse and Mental Health Services Administration National Household Survey on Drug Abuse and National Survey on Drug Use and Health; US Department of Education, National Center for Education Statistics, National Assessment of Educational Progress; and Youth Risk Behavior Surveillance System (eTables 1-8 in the Supplement). In addition, national data were gathered for American Indian or Alaska Native, Asian, Black, Latinx, and White subgroups. When multiple years of component data were pooled into a single estimate, we assigned that data point to the last year of the interval. In instances of missing data, we implemented simple linear interpolation whenever possible (ie, 2011 was imputed as the mean of 2010 and 2012), but there were instances wherein a different solution was required (eTable 1 in the Supplement).

### Index Construction

To aggregate the components into the Child and Adolescent Thriving Index 1.0, we used the following equation:
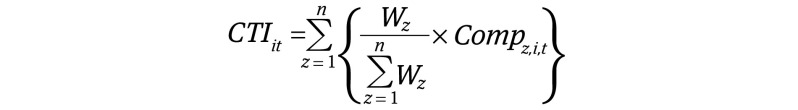
.We calculated the value of each component (z) for each geographic or racial and ethnic group (i) in each year (t) as the proportion of the population with the favorable outcome. Each component had a corresponding weight (W), which was identified through a data-driven process in a previous article^25^ (eTable 9 in the Supplement). We summed the weighted component scores for a given geographic location or race and ethnicity in a single year. The index has a possible range of 0 to 1, with 0 indicating minimum and 1 indicating maximum possible child and adolescent well-being at the population level.

### Statistical Analysis

To assess the robustness of the findings, we performed a Monte Carlo analysis with 10 000 simulations focusing on 2 sources of uncertainty: data quality and weight values. We calculated 90% credible intervals (the range of values this percentage of simulations covered). eAppendix, eTables 10 and 11, and eFigures 1 to 11 in the Supplement contain the methods and results of that analysis as well as several other subanalyses of uncertainty.

All statistical calculations were performed with Stata, version 17.0 (StataCorp LLC). Data were analyzed from June 7, 2021, to March 17, 2022.

## Results

The Child and Adolescent Thriving Index 1.0 was calculated from 11 components: non–low birth weight in neonates, preschool attendance in children aged 3 to 4 years, reading proficiency in fourth-grade students, math proficiency in eighth-grade students, food security in children younger than 18 years, general health status, nonobesity in high school students, nonsmoking in adolescents aged 12 to 17 years, non–marijuana use in adolescents aged 12 to 17 years, high school graduation in young adults aged 18 to 21 years, and nonarrest rate in children aged 10 to 17 years (eTable 1 in the Supplement). A total of 12 320 annual estimates covering these components over 20 years at the national, state, and racial and ethnic levels were used. Full data were available for 5 components: non–low birth weight in neonates, preschool attendance in children aged 3 to 4 years, food security in children younger than 18 years, general health status, and high school graduation in young adults aged 18 to 21 years. Two components, nonsmoking in adolescents aged 12 to 17 years and non–marijuana use in adolescents aged 12 to 17 years, had low levels of missing data (7.1% of their estimates), for which simple linear interpolation from surrounding years was used for imputation. Four other components (reading proficiency in fourth-grade students, math proficiency in eighth-grade students, nonobesity in high school students, and nonarrest rate in children aged 10-17 years) had much higher degrees of missing data (65.1%) because data were available biannually or a scalar transformation of a related measure was required. Across all 11 components, 25.9% of the required estimates were imputed.

### Trends in Well-being at the National Level

The Table shows results for the Child and Adolescent Thriving Index 1.0 and its components for 2000, 2010, and 2019. The overall index increased from 0.780 points in 2000 to 0.843 points in 2019, meaning that 28.6% of the gap in maximal well-being was closed. The increase was fairly even across both halves of the study period, with 53.6% of the increase occurring from 2010 to 2019.

**Table.** Descriptive Statistics of the Child and Adolescent Thriving Index 1.0 and Its Components for 2000, 2010, and 2019

	National score (state range)		
	2000	2010	2019
Overall index			
Child and Adolescent Thriving Index 1.0, points^a^	0.780 (0.735-0.812)	0.809 (0.763-0.847)	0.843 (0.808-0.871)
Index components, %			
Percentage of neonates not born with low birth weight of <5.5 lb^b^^,^^c^	92.4 (89.3-94.4)	91.9 (87.9-94.3)	91.7 (87.7-93.7)
Percentage of young children (aged 3-4 y) enrolled in preschool in the previous 3 mo^d^	49.3 (30.2-62.6)	47.7 (30.6-63.1)	49.2 (31.5-69.8)
Percentage of fourth-grade public school students proficient in reading^b^^,^^e^	29.0 (16.5-43.0)	32.0 (20.5-48.5)	34.0 (24.0-45.0)
Percentage of eighth-grade public school students proficient in math^b^^,^^e^	25.0 (9.0-39.0)	33.5 (17.0-51.5)	33.0 (21.0-47.0)
Percentage of children (aged <18 y) with food security^f^	93.5 (83.7-97.2)	95.1 (89.8-98.6)	97.9 (95.5-99.8)
Percentage of children (aged <18 y) not in fair or poor health^f^	97.5 (94.8-99.5)	97.7 (95.2-99.7)	98.2 (93.3-99.9)
Percentage of children in high school reporting no obesity^g^	90.2 (85.9-94.8)	87.6 (83.0-92.9)	84.5 (76.6-90.2)
Percentage of adolescents (aged 12-17 y) reporting no cigarette smoking in previous mo^h^	85.8 (77.6-91.1)	91.3 (86.5-94.1)	97.5 (94.4-98.8)
Percentage of adolescents (aged 12-17 y) reporting no marijuana use in previous y^h^	86.2 (78.9-90.6)	86.2 (80.5-90.7)	87.2 (78.9-91.4)
Percentage of young adults aged 18-21 y living in same state as previous y, with a high school degree^d^	76.4 (68.6-84.1)	82.1 (76.4-88.4)	86.1 (81.3-90.7)
Complement of (100 minus) arrests per 100 children, aged 10-17 y^i^	93.5 (83.7-97.2)	95.1 (89.8-98.6)	97.9 (95.5-99.7)

SI conversion factor: To convert pounds to kilograms, multiply by 0.45.

^a^ Potential range was 0 (indicating worst possible index) to 1 (indicating best possible index).

^b^ Data from KIDS COUNT data center.^26^

^c^ Data from 2000-2019 National Vital Statistics Reports, Centers for Disease Control and Prevention National Center for Health Statistics.

^d^ Based on authors’ calculations using 2000 US Census Bureau and 2001-2019 American Community Survey data; data retrieved from Ruggles et al.^27^

^e^ Data from 1998, 2002, and 2003-2019 National Assessment of Educational Progress, US Department of Education, National Center for Education Statistics.

^f^ Based on authors’ calculations using 2000-2019 Current Population Survey data; data retrieved from Ruggles et al.^27^

^g^ Data from 2001-2019 Youth Risk Behavior Surveillance System.

^h^ Data from combination of 2 surveys from the Substance Abuse and Mental Health Services Administration: 2000-2002 National Household Survey on Drug Abuse and 2003-2019 National Survey on Drug Use and Health.

^i^ Data from 2000-2012 and 2013-2019 Statistical Briefing Book, Office of Juvenile Justice and Delinquency Prevention.

**Figure 1.**
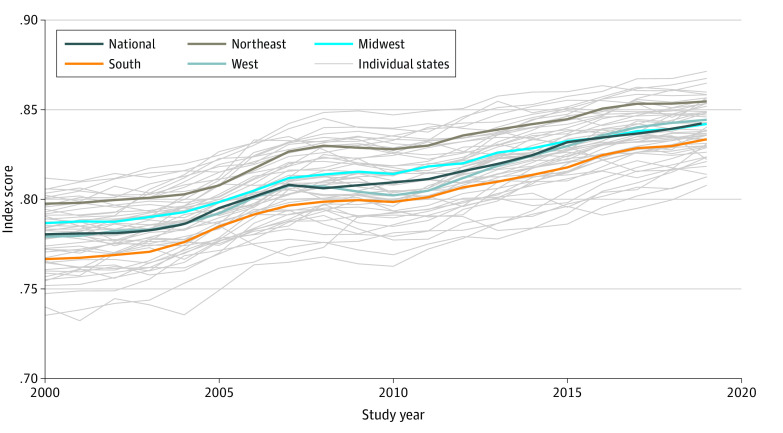
Trends in the Child and Adolescent Thriving Index 1.0 From 2000 to 2019 Index score ranges from 0 (indicating worst possible child and adolescent well-being) to 1 (indicating best possible child and adolescent well-being).

Figure 1 plots the national index scores from 2000 to 2019. As seen in the Table, national well-being scores increased steadily throughout the study period. From 2000 to 2007, the index improved by 0.004 points per year. The index stalled during the beginning of the Great Recession, declining by 0.001 points from 2007 to 2009 but returning to the same annual 0.004 points increase from 2009 to 2019. Results from the uncertainty analysis confirmed that the index unambiguously increased over the study period. When examining data quality and weight values, the 90% credible interval for the total increase in index scores from 2000 to 2019 ranged from 0.060 to 0.072 points. Analyses of other sources of uncertainty also showed increases in index score regardless of alteration to the method (eAppendix, eTables 10 and 11, and eFigures 1-11 in the Supplement).

### Trends in Well-being by State and Race and Ethnicity

Figure 1 also illustrates regional and state scores of the Child and Adolescent Thriving Index 1.0 from 2000 to 2019. Two findings were observed. First, in every single state, there was an increase in well-being scores over the study period. Second, there was slight evidence of convergence (eFigure 12 in the Supplement). eFigure 12 in the Supplement is illustrative of both of these observations. The y-axis shows the change in index score for all 50 states from 2000 to 2019, the minimum value of which is 0.042. The downward-sloping red line illustrates that states with a higher index score in 2000 had a smaller increase over the ensuing 2 decades. Poor-performing states, typically in the Appalachia and Sunbelt regions, narrowed their gap over time from higher-performing states, usually located in the Northeast and Upper Midwest. However, across all years, younger people in the Northeast tended to exhibit higher levels of well-being (vs national levels: +0.017 points in 2000 and +0.012 points in 2019), whereas those in the South typically fared worse (−0.021 points; vs Northeast levels: −0.031 points in 2000 and −0.021 points in 2019) (eFigure 13 and eTable 12 in the Supplement). Thus, convergence was minor compared with existing disparities.

**Figure 2.**
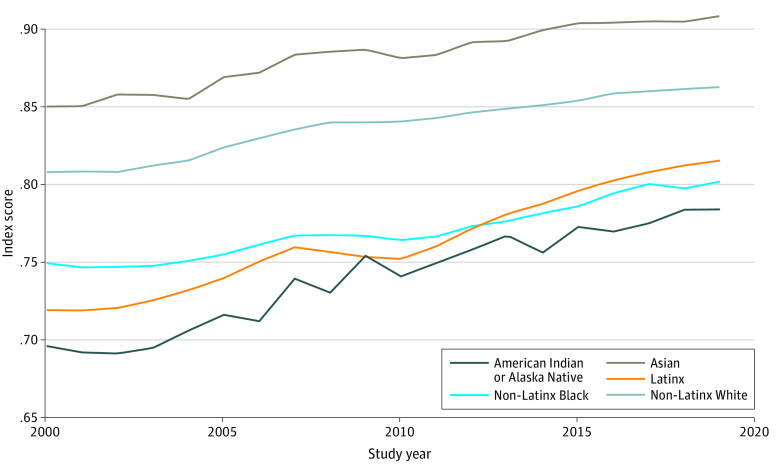
National-Level Child and Adolescent Thriving Index 1.0 by Race and Ethnicity From 2000 to 2019 Index score ranges from 0 (indicating worst possible child and adolescent well-being) to 1 (indicating best possible child and adolescent well-being).

Figure 2 shows the racial and ethnic disparities in national well-being over time. Asian (+0.070 points in 2000 and +0.066 points in 2019) and White (+0.027 points in 2000 and +0.020 points in 2019) youths had systematically higher levels of well-being compared with their peers from 2000 to 2019. Disparities between Black and White individuals were largely constant from 2000 to 2019 (from −0.059 points to −0.053 points). However, disparities between White youths and other racial and ethnic minority groups narrowed. For Latinx youths, the disparities narrowed by 46.6% from −0.089 points to −0.047 points. For American Indian or Alaska Native youths, the disparities narrowed by 29.4% from −0.112 points to −0.079 points. However, in both of these cases, most of the disparities were still present in 2019. These disparities were robust according to the analysis of uncertainty we conducted (eFigure 14 in the Supplement).

### Decomposing the Change in Index Scores of Well-being 

**Figure 3.**
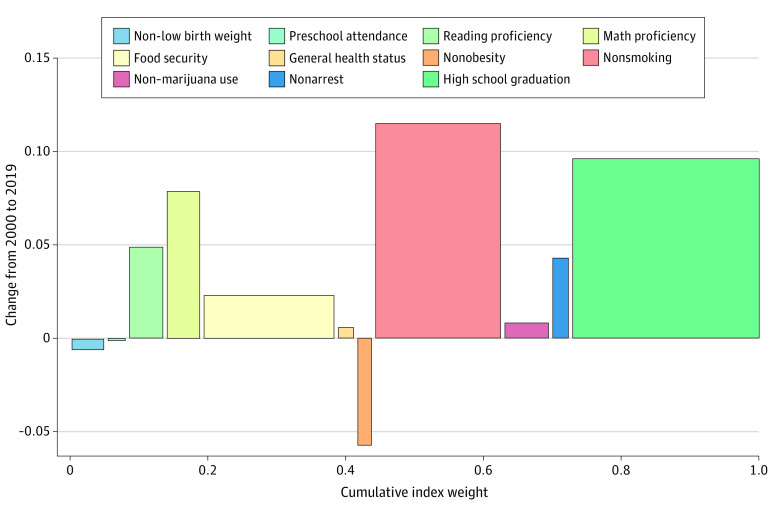
Decomposing Change in the National-Level Child and Adolescent Thriving Index 1.0 From 2000 to 2019 Outcomes on the x-axis are sorted according to life-course stage. The width of the bars indicates the index weight applied to the respective component, whereas the height indicates the change in population prevalence. Outcomes are arranged such that a positive y-axis value indicates an improvement.

Figure 3 decomposes the overall change in the national-level Child and Adolescent Thriving Index 1.0 scores from 2000 to 2019 by index component. The figure shows the index weight (represented by the width of the bar), the change in population prevalence (represented by the height of the bar), and the change in index units for each component (the product of the weight and prevalence, or the area of the bar). The higher index score was largely associated with changes in 2 components: increase in high school graduation rate (+0.028 units) and increase in nonsmoking rate in adolescents (+0.022 units), which amounted to 80.6% of the total increase. Six components had smaller increases in index scores (food security in children younger than 18 years: +0.005 units; general health status: less than +0.001 units; non–marijuana use in adolescents aged 12-17 years: +0.001 units; reading proficiency in fourth-grade students: +0.003 units; math proficiency in eighth-grade students: +0.004 units; nonarrest rate in children aged 10-17 years: +0.001 units), and 3 components (non–low birth weight in neonates: less than −0.001 units; preschool attendance in children aged 3-4 years: less than −0.001 units; and nonobesity in high school students: −0.001 units) exhibited a slight decline over the study period.

**Figure 4.**
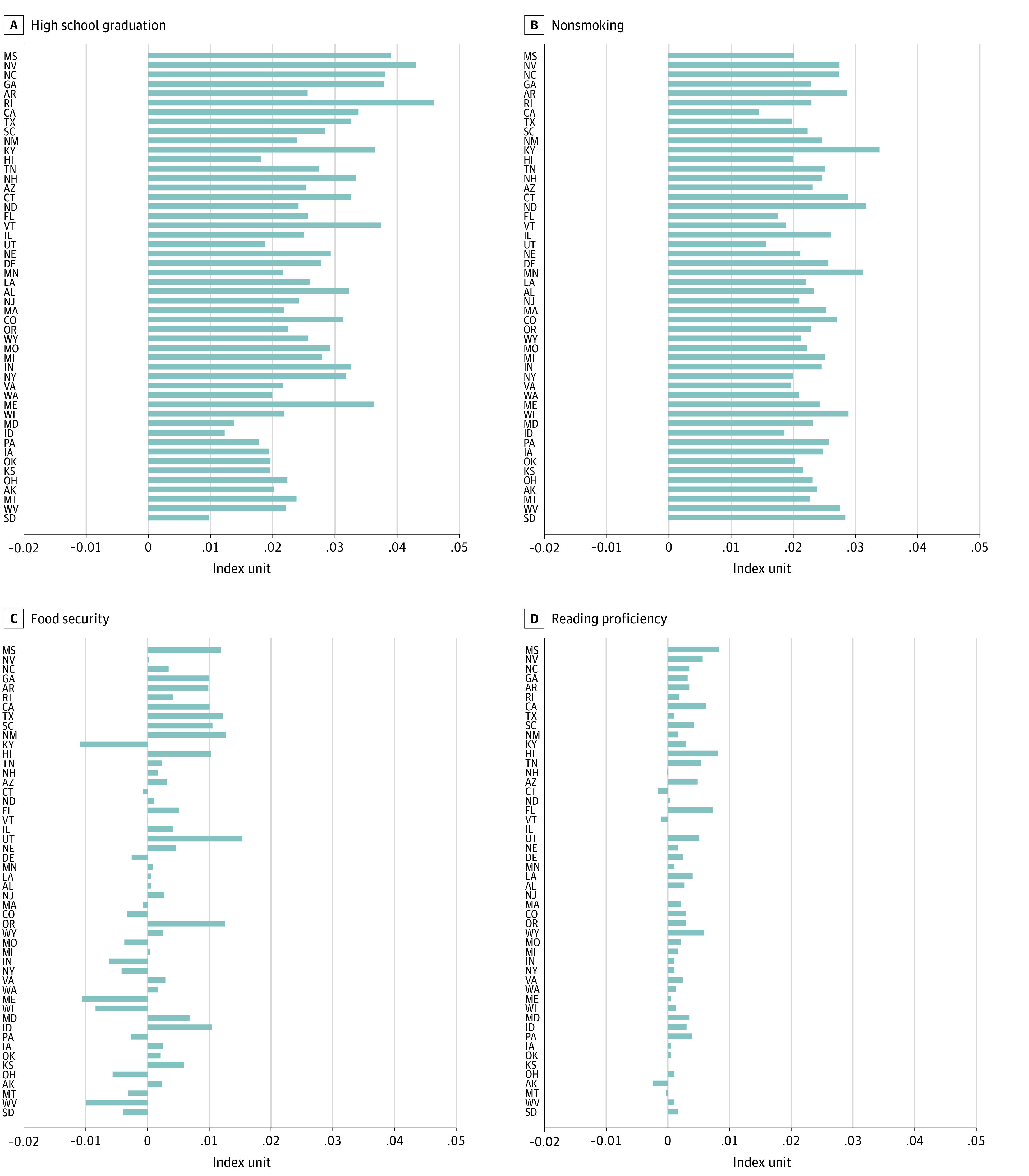
Select Contributors to Changes in the State-Level Child and Adolescent Thriving Index 1.0 From 2000 to 2019 States are sorted by their overall increase in the index score, with those listed at the top having higher scores (eg, Mississippi [MS] at 0.088 points) vs those listed near the bottom (eg, South Dakota [SD] at 0.042 points).

Figure 4 shows state-level scores for the 4 largest component changes. Large increases in high school graduation and nonsmoking rates occurred in all 50 states. This pattern was in contrast with the findings for food security and reading proficiency, wherein there were higher levels of variability. The coefficient of variation (the SD divided by the mean) was 288.5% for the changes in food security and 98.8% for the reading proficiency components compared with 29.0% for the high school graduation and 16.6% for the nonsmoking components (eTable 13 in the Supplement provides additional detail in state-level component increases in index scores from 2000 to 2019). eFigure 15 in the Supplement shows results for a similar component-level analysis by race and ethnicity.

## Discussion

In this analysis, we applied a new and more child-centric method to estimate 2 decades of child and adolescent well-being in the US. Trends in the Child and Adolescent Thriving Index 1.0 were clear: child and adolescent well-being index scores increased over the past 20 years, both nationally and across all 50 states. These results are consistent with those of previous studies.^14,15,16,17^

When looking at subnational estimates, we found some convergence over time: poor-performing states narrowed the well-being gap compared with higher-performing states. However, these poor-performing states still tended to perform worse in the most recent rankings.

Decomposing the Child and Adolescent Thriving Index 1.0 revealed 2 key components for understanding the increases in overall score: high school graduation and nonsmoking in adolescence. However, the way these components changed over the study period was somewhat different. High school graduation rates increased throughout the US, but some states performed better than others. Meanwhile, decreases in smoking rates among adolescents were both large and similar across the states.

Advancements in both indicators (high school graduation and nonsmoking) are examples of a successful public policy. For high school graduation, despite some concerns of data manipulation by state and local entities, there have been real improvements within the educational system.^28^ Additional hypotheses for such improvements include the long-term outcome of increasing preschool attendance, improved quality of elementary school education, and declining teenaged birth and arrest rates from the 1980s and 1990s.^28^ Expanding access to high-quality preschool, incentivizing higher teacher performance, and reducing class sizes are promising building blocks to these successes.^28^ For smoking in adolescence, years of public awareness campaigns may have helped reduce the appeal of cigarettes.^29^ Specific policies regulating cigarette advertising, increasing sales taxes, and raising the legal smoking age may have also been effective.^30^

Although food security had a similar index weight as nonsmoking rates, the vast differences across states meant that the realized gains in the overall national index score through this pathway were smaller. The variation in state-level changes in food security suggests that policy makers have real power to change this aspect of well-being. Bolstering programs, such as the Supplemental Nutrition Assistance Program and Women, Infants, and Children Program, by expanding eligibility and benefit generosity as well as removing administrative burden to accessing these programs are key changes that could have substantial consequences.^31,32^

We anticipated finding patterns of convergence for racial and ethnic minority groups compared with White groups based on results from earlier periods.^18^ However, from 2000 to 2019, there were improvements in inequities between American Indian or Alaska Native and White youths and between Latinx and White youths, but the disparities between Black and White children and adolescents remained similar. Larger forces of institutional racism (eg, housing segregation, access to credit markets, and different treatment by the criminal justice system) and structural racism (ie, the way these forces interact with and reinforce one another) should be understood as the root causes for these inequities in well-being.^5,33^

The trends of steady increase in the Child and Adolescent Thriving Index 1.0, which continues throughout 2000 to 2019, contrast with worsening levels of mental distress and subjective well-being that began around 2012.^34,35^ This divergence of composite index scores with mental health indicators is a recent development: estimates from an earlier index found a correlation of 0.65 with national levels of life satisfaction among high school students from 1975 to 2003.^16^ The abruptness and timing of the divergence support the theory that widespread introduction of social media, a construct not captured by the Child and Adolescent Thriving Index 1.0, may have adverse implications for population mental health.^36^ Although more work is required to confirm this hypothesis, the discrepancy between the index and more direct measures of mental health highlights the importance of continuing to develop population data systems to better reflect important aspects of younger peoples’ lives. Adding measures such as good and bad experiences with social media, the quality of relationships with friends and family, and levels of loneliness is an essential step for the next generation of population-level well-being measurement.^37^

### Limitations

This study has several limitations. The accuracy of a composite index is always subject to the quality of the underlying component data. The choice of how to measure high school graduation is an example.^38,39^ To ensure that the results were robust to the preferred specification, we performed a comparison. eFigure 16 in the Supplement confirmed that national findings were essentially the same when adopting an alternative measure of high school graduation, although there were some discrepancies across states. Several other components were subject to high rates of missing data at the subnational level (nonobesity, test scores, and nonarrest rate), but these measures had a relatively smaller role in the index construction (12.7% of the cumulative weights), meaning that any measurement error was unlikely to change the analysis of inequities.

The choice of component measures for a composite index required some subjective decisions even when assisted with a data-driven process. For example, we did not directly include measures of mental health or subjective well-being as components because doing so would place those measures on both the left and right sides of a regression in the original method analysis.^25^ The approach we used assumed that these constructs should be well reflected by the weights assigned to the objective components through those models, but they were still imperfect proxies at best. Other objective measures that may be important to well-being, such as immunization rates, bullying, socioemotional health, and relationship quality, were not included because estimates were unavailable at the national and/or state levels or they were not collected in the original study used to derive the index methods.

Composite indices are measurement models. The relative weights and importance of the components in well-being should not be interpreted as causal. For example, worsening low birth weight and obesity rates did not have substantial implications for the index score largely because these components had smaller relative weights, which does not mean these measures are unimportant aspects of well-being. Improving both outcomes could be associated with future increases in other index components, such as high school graduation and nonsmoking rates.

These indices combine all stages of childhood and adolescence into 1 measure. Although this simplification was intended to facilitate more widespread adoption of the measure, stakeholders may also want measures that are focused on specific developmental periods. The Healthy and Ready to Learn program and Early Development Instrument are 2 such examples geared toward well-being in early childhood.^40,41^

## Conclusions

A deeper understanding of younger peoples’ well-being is critical. Indices of child and adolescent well-being hold great promise for achieving this aim. This study presented population-level findings from a new index, the Child and Adolescent Thriving Index 1.0, which has been validated against existing measures. Although child and adolescent well-being improved significantly from 2000 to 2019, there is substantial room for improvement. Inequities across states and racial and ethnic populations must be addressed, with a focus on economic inequality and structural racism, systems that shape the contexts in which younger people play, learn, and grow. Future work should build on these descriptive patterns by using the new index to evaluate public policy, such as assessing which policy types (eg, economic, social, health care, housing, or education) are associated with higher levels of well-being. Continued progress in this area is needed to fulfill the public health mission of providing the conditions under which all people can be healthy.

## Notes

Supplement. **eTable 1.** Data Sources for Index Components **eTable 2.** Smoking Component Data Standard Errors by Year **eTable 3.** Marijuana Component Data Standard Errors by Year **eTable 4.** Obesity Component Data Standard Errors by Year **eTable 5.** Fair/Poor Health Component Data Sample Sizes by Year **eTable 6.** Preschool Component Data Sample Sizes by Year **eTable 7.** High School Graduation Component Data Sample Sizes by Year **eTable 8.** Food Security Data Sample Sizes by Year **eTable 9.** Child and Adolescent Thriving Index 1.0 Weights **eAppendix.** Uncertainty Analysis and Monte Carlo Simulation Methodology **eFigure 1.** National Child and Adolescent Thriving Index 1.0, 2000-2019 **eFigure 2.** Robustness of Selection of Indicators Comparing to Alternate Variable Selection Results **eFigure 3.** Robustness of Weighting Scheme Comparing to KIDS COUNT (Equally Weighted Components) **eFigure 4.** Robustness of Data Standardization Methods Comparing With Version Where Components Are Normalized **eFigure 5.** Robustness of Aggregation Methods Comparing With Substitutability Adjustment **eFigure 6.** 90% Credible Intervals of State Rankings for Select Years With Various Forms of Uncertainty **eTable 10.** Probability of Being Within 5 Ranks of Calculated Ranking by Source of Uncertainty, 2000-2019 **eTable 11.** Margin of Error Analysis: Number of Ranks Above or Below Calculated Rank, For Which There Is a 90% Probability of the True Rank, by Source of Uncertainty, 2000-2019 **eFigure 7.** Robustness of Selection of Indicators Comparison With Contextual Variable Index **eFigure 8.** Robustness of Selection of Indicators Comparison With Alternative Model Selection Procedure **eFigure 9.** Robustness of Weighting Scheme Comparing to KIDS COUNT Ranks (Equally Weighted Components) **eFigure 10.** Robustness of Data Standardization Methods Comparing With Version Where Components Are Normalized **eFigure 11.** Robustness of Aggregation Methods Comparing With Substitutability Adjustment **eFigure 12.** Convergence in State-Level Child and Adolescent Thriving Index 1.0, 2000-2019 **eFigure 13.** State-Level Child and Adolescent Thriving Index 1.0, Select Years **eTable 12.** Rank Differences Across Annie E. Casey KIDS COUNT Index (AEC) and Child and Adolescent Thriving Index 1.0 (CATI-1.0) **eFigure 14.** Disparities with White Children, National Child and Adolescent Thriving Index 1.0, 2000-2019 **eTable 13.** Pairwise Correlation Analysis of Changes in State-Level Index Component Values from 2000-2019 **eFigure 15.** Decomposing Change in National Child and Adolescent Thriving Index 1.0 by Race/Ethnicity **eFigure 16.** Comparison When Using ACGR Measure
eReferences
