# Supplementary material for: Trends in Measures of Child and Adolescent Well-being in the US From 2000 to 2019
Source: JAMA Netw Open. 2022 Oct 26;5(10):e2238582. doi: 10.1001/jamanetworkopen.2022.38582 (PMC9606848; doi:10.1001/jamanetworkopen.2022.38582)
Supplement: Supplement. — eTable 1. Data Sources for Index Components eTable 2. Smoking Component Data Standard Errors by Year eTable 3. Marijuana Component Data Standard Errors by Year eTable 4. Obesity Component Data Standard Errors by Year eTable 5. Fair/Poor Health Component Data Sample Sizes by Year eTable 6. Preschool Component Data Sample Sizes by Year eTable 7. High School Graduation Component Data Sample Sizes by Year eTable 8. Food Security Data Sample Sizes by Year eTable 9. Child and Adolescent Thriving Index 1.0 Weights eAppendix. Uncertainty Analysis and Monte Carlo Simulation Methodology eFigure 1. National Child and Adolescent Thriving Index 1.0, 2000-2019 eFigure 2. Robustness of Selection of Indicators Comparing to Alternate Variable Selection Results eFigure 3. Robustness of Weighting Scheme Comparing to KIDS COUNT (Equally Weighted Components) eFigure 4. Robustness of Data Standardization Methods Comparing With Version Where Components Are Normalized eFigure 5. Robustness of Aggregation Methods Comparing With Substitutability Adjustment eFigure 6. 90% Credible Intervals of State Rankings for Select Years With Various Forms of Uncertainty eTable 10. Probability of Being Within 5 Ranks of Calculated Ranking by Source of Uncertainty, 2000-2019 eTable 11. Margin of Error Analysis: Number of Ranks Above or Below Calculated Rank, For Which There Is a 90% Probability of the True Rank, by Source of Uncertainty, 2000-2019 eFigure 7. Robustness of Selection of Indicators Comparison With Contextual Variable Index eFigure 8. Robustness of Selection of Indicators Comparison With Alternative Model Selection Procedure eFigure 9. Robustness of Weighting Scheme Comparing to KIDS COUNT Ranks (Equally Weighted Components) eFigure 10. Robustness of Data Standardization Methods Comparing With Version Where Components Are Normalized eFigure 11. Robustness of Aggregation Methods Comparing With Substitutability Adjustment eFigure 12. Convergence in State-Level Child and Adolescent Thriving Index [file jamanetwopen-e2238582-s001.pdf]

Supplemental Online Content

Anderson NW, Eisenberg D, Halfon N, Markowitz A, Moore KA, Zimmerman FJ. Trends in measures of child and adolescent well-being in the US from 2000 to 2019. *JAMA Netw Open*. 2022;5(10):e2238582. doi:10.1001/jamanetworkopen.2022.38582

eTable 1. Data Sources for Index Components
eTable 2. Smoking Component Data Standard Errors by Year
eTable 3. Marijuana Component Data Standard Errors by Year
eTable 4. Obesity Component Data Standard Errors by Year
eTable 5. Fair/Poor Health Component Data Sample Sizes by Year
eTable 6. Preschool Component Data Sample Sizes by Year
eTable 7. High School Graduation Component Data Sample Sizes by Year
eTable 8. Food Security Data Sample Sizes by Year
eTable 9. Child and Adolescent Thriving Index 1.0 Weights
eAppendix. Uncertainty Analysis and Monte Carlo Simulation Methodology
eFigure 1. National Child and Adolescent Thriving Index 1.0, 2000-2019
eFigure 2. Robustness of Selection of Indicators Comparing to Alternate Variable Selection Results
eFigure 3. Robustness of Weighting Scheme Comparing to KIDS COUNT (Equally Weighted Components)
eFigure 4. Robustness of Data Standardization Methods Comparing With Version Where Components Are Normalized
eFigure 5. Robustness of Aggregation Methods Comparing With Substitutability Adjustment
eFigure 6. 90% Credible Intervals of State Rankings for Select Years With Various Forms of Uncertainty
eTable 10. Probability of Being Within 5 Ranks of Calculated Ranking by Source of Uncertainty, 2000-2019
eTable 11. Margin of Error Analysis: Number of Ranks Above or Below Calculated Rank, For Which There Is a 90% Probability of the True Rank, by Source of Uncertainty, 2000-2019
eFigure 7. Robustness of Selection of Indicators Comparison With Contextual Variable Index
eFigure 8. Robustness of Selection of Indicators Comparison With Alternative Model Selection Procedure
eFigure 9. Robustness of Weighting Scheme Comparing to KIDS COUNT Ranks (Equally Weighted Components)
eFigure 10. Robustness of Data Standardization Methods Comparing With Version Where Components Are Normalized
eFigure 11. Robustness of Aggregation Methods Comparing With Substitutability Adjustment
eFigure 12. Convergence in State-Level Child and Adolescent Thriving Index 1.0, 2000-2019
eFigure 13. State-Level Child and Adolescent Thriving Index 1.0, Select Years
eTable 12. Rank Differences Across Annie E. Casey KIDS COUNT Index (AEC) and Child and Adolescent Thriving Index 1.0 (CATI-1.0)
eFigure 14. Disparities with White Children, National Child and Adolescent Thriving Index 1.0, 2000-2019

© 2022 Anderson NW et al. *JAMA Network Open.*

eTable 13. Pairwise Correlation Analysis of Changes in State-Level Index Component Values from 2000-2019
eFigure 15. Decomposing Change in National Child and Adolescent Thriving Index 1.0 by Race/Ethnicity
eFigure 16. Comparison When Using ACGR Measure
eReferences

This supplemental material has been provided by the authors to give readers additional information about their work.

eTable 1. Data Sources for Index Components

Component	Source	Data Availability	Imputation Notes
Percent of babies Born with Low Birthweight (less than 5.5 pounds)	2000-2019: Centers for Disease Control and Prevention, National Center for Health Statistics, National Vital Statistics Reports *	Mean: All Years<br>SE: Population Measure	
Percent of young children (3-4) not enrolled in school in the previous three months	2000: US Census<br>2001-2019: American Community Survey downloaded from Integrated Public Use Microdata Series (IPUMS-ACS)	Mean: All years<br>SE: All years	
Percentage of 4th grade public school students not proficient in reading	1998 & 2002, 2003-2019 (odd years): U.S. Department of Education, National Center for Education Statistics, National Assessment of Educational Progress (NAEP).*	Mean: All values in years listed except following for 1998: AK, ID, IL, IN, ND, NE, NJ, OH, PA, SD, and VT ; 2002: AK, CO, IL, NH, NJ, SD, and WI.<br>SE: Population Measure	Need 1998 values for imputation.<br>Interpolate 1998 using 2002-2005 trend if 2002 is present.<br>Interpolate 2002 using 1998-2003 trend if 1998 is present.<br>Interpolate 1998 and 2002 using 2003-2005 trend if neither1998 nor 2002 is present.<br>Lastly interpolate all other values (including even years) as missing values
Percentage of 8th grade public school students not proficient in math	2000, 2003-2019 (odd years): U.S. Department of Education, National Center for Education Statistics, National Assessment of Educational Progress (NAEP).*	Mean: All values in years listed except following for 2000: AK, CO, DE, FL, IA, NH, NJ, PA, SD, WA, & WI<br>SE: Population Measure	Interpolate missing 2000 values using 2003-2005 trend.<br>Then interpolate all other values (including even years) as missing values
Percent of children (Under 18) with food insecurity	2000-2019: Current Population Survey downloaded from Integrated Public Use Microdata Series (IPUMS-CPS)	Mean: All Years<br>SE: All Years	
Percent of children (0-17) reported not in fair or poor health by their parent/guardian	2000-2019: Current Population Survey	Mean: All Years<br>SE: All Years	
Percent of children in high school reporting obesity	2001-2019 (odd years): Youth Risk Behavior Surveillance System State Estimates (YRBSS)	Mean: All years listed except for following: 2001 (AK, AZ, CA, CO, CT, GA, HI, IL, IN, IA, KS, KY, LA, MD, MN, NE, NV, NH, NM, NY, OH, OK, OR, PA, SC, TN, VA, WA, WV) ; 2003 (AR, CA, CO, CT, HI, IN, IA, KS, LA, MD, MN, NV, NJ, NM, OR, PA, SC, TX, VA, WA) ; 2005 (AK, CA, IL, LA, MN, MI, NV, OR, PA, VA, WA) ; 2007 (AL, CA, CO, MN, NE, NJ, OR, PA,	Interpolate missing values for 2017 and 2019 using NSCH estimate.<br>Then interpolate missing odd years using average values of data from prior and later years where possible.<br>Then interpolate remaining missing odd years using the national trend as the basis.<br>Lastly, interpolate even years using average of surrounding odd years,

© 2022 Anderson NW et al. *JAMA Network Open.*

		VA, WA) ; 2009 (CA, IA, MN, NE, OH, OR, VA, WA) ; 2011 (CA, MN, MO, NV, OR, PA, WV) ; 2013 (CA, CO, IN, IA, MN, OR, PA, WA) ; 2015 (CO, GA, IA, KS, LA, MN, NJ, OH, OR, TX, UT, WA, WI) ; 2017 (AL, GA, IN, MN, MI, NJ, OH, OR, SD, WA, WY) ; 2019 (DE, IN, MN, OR, WA, WY)<br>SE: Available whenever mean is	
Percent of children (12-17) reporting smoking in prior month	2000-2002: Substance Abuse and Mental Health Services Administration - National Household Survey on Drug Abuse (SAMHSA-NHSDA)<br>2003-2019: Substance Abuse and Mental Health Services Administration - National Survey on Drug Use and Health State Estimates (SAMHSA- NSDUH)	Mean: All Years (except Asian in 2019).<br>SE: All years except 2000-2001 national estimates; 2001 race/ethnicity estimates	Impute 2019 Asian as same as 2018 (suppressed because very low value [0.9% in 2018]). Impute 2000-2002 national SEs as average of 2003-2005 values.
Percent of children (12-17) reporting using marijuana in prior year	2001: Substance Abuse and Mental Health Services Administration - National Household Survey on Drug Abuse (SAMHSA-NHSDA)2003-2019: Substance Abuse and Mental Health Services Administration - National Survey on Drug Use and Health State Estimates (SAMHSA- NSDUH)	Mean: All years except 2000-2002 minus 2001 national estimate. All race/ethnicities in 2001, plus AIAN in 2000, 2004, 2006, and 2007.<br>SE: All years except 2000-2002.	Impute all missing geography estimates using national 2001 -2003 trend.<br>For AIAN, impute using trend for 2000, and other years are interpolated as average of surrounding years.<br>Impute 2000-2002 SEs as average of 2003-2005 values.
Percentage of resident (didn't move states) young adults (18-21) with a high school degree	2000-2019: American Community Survey downloaded from Integrated Public Use Microdata Series (IPUMS-ACS)	Mean: All years<br>SE: N/A	
Arrest rate per 100,000 children	2000-2012: Arrest Types – Violent crime index, Property crime index, Drug Abuse, and Weapons<br>2013-2019: Aggravated assault, Robbery, Larceny, Drug Abuse, and Weapons<br>All data is from Office of Juvenile Justice and Delinquency Prevention.<br>Since there is no information on Latinx status for this data, we do not collect it for race/ethnicity, and modify the index formula to omit the arrests component	Mean: A similar measure (see discussion of multiplicative scalar in next section) was available all years except – HI (2007, 2011), IA (2018), KS (2000, 2001), MT (2004, 2006), NV (2003), WI (2000)<br>SE: Population Measure	Interpolate using surrounding years if missing. Interpolate using linear trend if no prior years of data.<br>Because only certain types of juvenile arrests are listed by state, we apply a scalar using the national juvenile crime rate (from Bureau of Justice Statistics) to all state estimates.

Sources: IPUMS-CPS ; IPUMS-ACS ; SAMHSA -NSDUH '03-'19 ; SAMHSA -NHSDA '00, '01, and '02 ; YRBSS ; NSCH ; NAEP ; OJJDP

Notes: Color key: green is full; yellow is mostly; red is every other year at best. Measures here include those in the Child and Adolescent Thriving Index 1.0, as well as author's version of the KIDS COUNT Index. The KIDS COUNT Index is based on the most recent set of measures used (with the exception of illicit drug use which was replaced by overweight and obesity in the 2020 chartbook).

* = Originally extracted from KIDS COUNT Data Center. SE = Standard Error. "N/A" for standard error availability indicates that measure is only for KIDS COUNT index, which does not have uncertainty modeled. "Population Measure" for standard error availability indicates the data is for the entire population and therefore shouldn't have a standard error estimate – however, to accommodate for some error we take the corresponding standard error from the CPS pre-k estimate and divide by 6.

© 2022 Anderson NW et al. *JAMA Network Open.*

eTables 2 through 8 show the relative uncertainty of estimates by geography / race ethnicity. We prefer to show sample sizes where possible, but in some instances rely on standard errors when data was extracted from an online database, instead of directly calculated by the research team. Measures that are collected for the entire target population (low birthweight, math proficiency, reading proficiency, and arrests) are not reported here.

eTable 2. Smoking Component Data Standard Errors by Year

	'00	'01	'02	'03	'04	'05	'06	'07	'08	'09	'10	'11	'12	'13	'14	'15	'16	'17	'18	'19
**USA**	IMP	IMP	IMP	0.0021	0.0021	0.0020	0.0019	0.0019	0.0018	0.0018	0.0019	0.0017	0.0016	0.0015	0.0014	0.0014	0.0012	0.0012	0.0012	0.0011
**State**																				
AL	0.0127	0.0116	0.0134	0.0114	0.0111	0.0107	0.0096	0.0095	0.0088	0.0088	0.0083	0.0083	0.0080	0.0067	0.0063	0.0061	0.0053	0.0054	0.0067	0.0049
AK	0.0126	0.0129	0.0139	0.0111	0.0116	0.0104	0.0092	0.0083	0.0081	0.0085	0.0088	0.0084	0.0080	0.0068	0.0068	0.0073	0.0082	0.0067	0.0061	0.0055
AZ	0.0118	0.0112	0.0147	0.0118	0.0114	0.0106	0.0096	0.0096	0.0084	0.0082	0.0101	0.0093	0.0072	0.0058	0.0056	0.0049	0.0038	0.0031	0.0036	0.0030
AR	0.0136	0.0119	0.0157	0.0125	0.0118	0.0110	0.0108	0.0116	0.0101	0.0100	0.0096	0.0093	0.0083	0.0072	0.0076	0.0068	0.0058	0.0053	0.0066	0.0050
CA	0.0041	0.0051	0.0069	0.0051	0.0054	0.0058	0.0048	0.0044	0.0043	0.0047	0.0047	0.0047	0.0041	0.0035	0.0032	0.0029	0.0026	0.0021	0.0020	0.0016
CO	0.0137	0.0126	0.0160	0.0117	0.0116	0.0103	0.0088	0.0085	0.0086	0.0086	0.0088	0.0088	0.0079	0.0065	0.0070	0.0054	0.0041	0.0040	0.0055	0.0048
CT	0.0142	0.0137	0.0161	0.0121	0.0117	0.0110	0.0088	0.0086	0.0078	0.0079	0.0086	0.0084	0.0070	0.0056	0.0053	0.0054	0.0043	0.0032	0.0030	0.0027
DE	0.0123	0.0127	0.0150	0.0114	0.0112	0.0101	0.0087	0.0082	0.0083	0.0086	0.0088	0.0085	0.0079	0.0070	0.0067	0.0055	0.0039	0.0032	0.0042	0.0032
FL	0.0067	0.0067	0.0088	0.0063	0.0058	0.0057	0.0053	0.0055	0.0051	0.0049	0.0050	0.0046	0.0040	0.0037	0.0037	0.0032	0.0029	0.0027	0.0032	0.0026
GA	0.0095	0.0100	0.0136	0.0110	0.0108	0.0097	0.0085	0.0085	0.0081	0.0080	0.0089	0.0095	0.0083	0.0062	0.0049	0.0040	0.0041	0.0038	0.0037	0.0031
HI	0.0112	0.0117	0.0133	0.0088	0.0097	0.0081	0.0075	0.0073	0.0072	0.0084	0.0086	0.0080	0.0072	0.0064	0.0059	0.0043	0.0040	0.0036	0.0037	0.0028
ID	0.0111	0.0110	0.0139	0.0109	0.0105	0.0098	0.0086	0.0083	0.0075	0.0079	0.0083	0.0085	0.0078	0.0067	0.0065	0.0062	0.0054	0.0051	0.0054	0.0041
IL	0.0069	0.0066	0.0085	0.0062	0.0059	0.0056	0.0054	0.0056	0.0054	0.0054	0.0053	0.0047	0.0043	0.0039	0.0042	0.0040	0.0038	0.0031	0.0029	0.0024
IN	0.0120	0.0118	0.0156	0.0117	0.0104	0.0104	0.0089	0.0095	0.0093	0.0088	0.0087	0.0079	0.0086	0.0075	0.0070	0.0073	0.0061	0.0054	0.0050	0.0047
IA	0.0132	0.0124	0.0163	0.0118	0.0112	0.0107	0.0099	0.0090	0.0085	0.0088	0.0097	0.0096	0.0084	0.0074	0.0072	0.0068	0.0054	0.0045	0.0047	0.0056
KS	0.0119	0.0123	0.0159	0.0119	0.0127	0.0116	0.0096	0.0094	0.0093	0.0094	0.0093	0.0094	0.0078	0.0062	0.0062	0.0057	0.0046	0.0041	0.0041	0.0035
KY	0.0152	0.0151	0.0154	0.0131	0.0135	0.0132	0.0115	0.0122	0.0110	0.0102	0.0099	0.0106	0.0103	0.0083	0.0077	0.0079	0.0069	0.0063	0.0077	0.0066
LA	0.0116	0.0121	0.0146	0.0121	0.0102	0.0089	0.0085	0.0095	0.0081	0.0079	0.0084	0.0082	0.0073	0.0080	0.0075	0.0064	0.0063	0.0049	0.0049	0.0047
ME	0.0123	0.0122	0.0143	0.0108	0.0120	0.0123	0.0102	0.0094	0.0090	0.0086	0.0092	0.0088	0.0082	0.0071	0.0070	0.0065	0.0069	0.0065	0.0068	0.0058
MD	0.0123	0.0115	0.0127	0.0097	0.0095	0.0094	0.0082	0.0076	0.0069	0.0076	0.0078	0.0066	0.0062	0.0053	0.0049	0.0047	0.0040	0.0033	0.0037	0.0029
MA	0.0123	0.0119	0.0151	0.0105	0.0100	0.0096	0.0088	0.0086	0.0072	0.0073	0.0077	0.0079	0.0073	0.0060	0.0055	0.0049	0.0038	0.0033	0.0043	0.0034
MI	0.0071	0.0069	0.0087	0.0063	0.0062	0.0060	0.0057	0.0055	0.0054	0.0055	0.0052	0.0051	0.0047	0.0044	0.0044	0.0043	0.0041	0.0035	0.0036	0.0032
MN	0.0136	0.0135	0.0162	0.0121	0.0109	0.0105	0.0101	0.0098	0.0086	0.0084	0.0083	0.0085	0.0087	0.0069	0.0060	0.0053	0.0049	0.0045	0.0048	0.0044
MS	0.0125	0.0116	0.0127	0.0109	0.0106	0.0098	0.0083	0.0082	0.0078	0.0088	0.0095	0.0088	0.0085	0.0082	0.0073	0.0064	0.0058	0.0054	0.0062	0.0057
MO	0.0125	0.0118	0.0171	0.0132	0.0129	0.0120	0.0101	0.0098	0.0092	0.0092	0.0104	0.0105	0.0084	0.0079	0.0086	0.0074	0.0066	0.0058	0.0058	0.0049
MT	0.0130	0.0128	0.0153	0.0119	0.0123	0.0116	0.0103	0.0097	0.0088	0.0093	0.0107	0.0106	0.0095	0.0072	0.0068	0.0071	0.0073	0.0065	0.0070	0.0072
NE	0.0126	0.0129	0.0184	0.0128	0.0114	0.0112	0.0092	0.0094	0.0084	0.0085	0.0086	0.0086	0.0077	0.0066	0.0068	0.0060	0.0046	0.0043	0.0041	0.0041
NV	0.0136	0.0132	0.0163	0.0115	0.0109	0.0098	0.0090	0.0088	0.0087	0.0083	0.0081	0.0079	0.0078	0.0064	0.0061	0.0053	0.0044	0.0036	0.0044	0.0033
NH	0.0125	0.0134	0.0176	0.0115	0.0106	0.0100	0.0090	0.0087	0.0081	0.0086	0.0096	0.0105	0.0086	0.0069	0.0070	0.0063	0.0058	0.0054	0.0055	0.0046

© 2022 Anderson NW et al. *JAMA Network Open.*

NJ	0.0097	0.0098	0.0140	0.0105	0.0107	0.0100	0.0087	0.0080	0.0070	0.0072	0.0077	0.0085	0.0072	0.0055	0.0054	0.0039	0.0034	0.0031	0.0032	0.0024
NM	0.0128	0.0150	0.0162	0.0120	0.0117	0.0109	0.0099	0.0103	0.0088	0.0090	0.0094	0.0092	0.0092	0.0082	0.0067	0.0051	0.0039	0.0038	0.0050	0.0052
NY	0.0069	0.0064	0.0089	0.0059	0.0060	0.0059	0.0052	0.0049	0.0050	0.0049	0.0052	0.0046	0.0043	0.0037	0.0037	0.0037	0.0030	0.0024	0.0026	0.0021
NC	0.0113	0.0119	0.0176	0.0123	0.0116	0.0117	0.0088	0.0088	0.0087	0.0082	0.0082	0.0085	0.0075	0.0065	0.0060	0.0045	0.0041	0.0036	0.0040	0.0039
ND	0.0140	0.0153	0.0176	0.0128	0.0129	0.0104	0.0094	0.0099	0.0095	0.0087	0.0096	0.0092	0.0091	0.0078	0.0078	0.0077	0.0064	0.0057	0.0063	0.0055
OH	0.0076	0.0072	0.0089	0.0067	0.0066	0.0063	0.0058	0.0060	0.0055	0.0055	0.0060	0.0055	0.0050	0.0045	0.0047	0.0051	0.0045	0.0042	0.0046	0.0041
OK	0.0126	0.0132	0.0155	0.0119	0.0130	0.0124	0.0108	0.0113	0.0101	0.0103	0.0098	0.0101	0.0086	0.0069	0.0081	0.0071	0.0055	0.0049	0.0060	0.0059
OR	0.0123	0.0114	0.0139	0.0101	0.0098	0.0101	0.0094	0.0086	0.0084	0.0090	0.0091	0.0077	0.0073	0.0065	0.0067	0.0060	0.0049	0.0044	0.0046	0.0045
PA	0.0073	0.0066	0.0093	0.0067	0.0064	0.0063	0.0060	0.0064	0.0060	0.0063	0.0061	0.0057	0.0051	0.0047	0.0048	0.0043	0.0036	0.0035	0.0037	0.0033
RI	0.0123	0.0127	0.0166	0.0119	0.0114	0.0111	0.0093	0.0094	0.0083	0.0080	0.0085	0.0084	0.0081	0.0062	0.0058	0.0052	0.0044	0.0037	0.0038	0.0032
SC	0.0124	0.0116	0.0135	0.0107	0.0102	0.0098	0.0094	0.0096	0.0080	0.0085	0.0084	0.0094	0.0088	0.0071	0.0058	0.0054	0.0055	0.0049	0.0067	0.0055
SD	0.0142	0.0140	0.0188	0.0142	0.0138	0.0122	0.0108	0.0101	0.0093	0.0097	0.0095	0.0098	0.0091	0.0078	0.0076	0.0073	0.0078	0.0070	0.0062	0.0057
TN	0.0131	0.0131	0.0161	0.0117	0.0110	0.0106	0.0099	0.0101	0.0091	0.0083	0.0083	0.0087	0.0084	0.0070	0.0068	0.0058	0.0057	0.0052	0.0057	0.0055
TX	0.0058	0.0065	0.0082	0.0065	0.0060	0.0057	0.0054	0.0054	0.0052	0.0052	0.0053	0.0048	0.0041	0.0040	0.0037	0.0034	0.0029	0.0024	0.0027	0.0027
UT	0.0113	0.0112	0.0124	0.0095	0.0087	0.0094	0.0082	0.0072	0.0067	0.0065	0.0077	0.0070	0.0072	0.0066	0.0065	0.0049	0.0041	0.0037	0.0036	0.0033
VT	0.0119	0.0124	0.0150	0.0116	0.0114	0.0111	0.0098	0.0094	0.0086	0.0080	0.0092	0.0099	0.0088	0.0077	0.0075	0.0069	0.0070	0.0065	0.0075	0.0064
VA	0.0104	0.0096	0.0158	0.0119	0.0106	0.0104	0.0090	0.0091	0.0083	0.0083	0.0083	0.0069	0.0065	0.0063	0.0054	0.0042	0.0040	0.0037	0.0039	0.0038
WA	0.0108	0.0111	0.0138	0.0101	0.0099	0.0091	0.0083	0.0085	0.0082	0.0079	0.0077	0.0082	0.0077	0.0067	0.0063	0.0048	0.0043	0.0039	0.0052	0.0040
WV	0.0149	0.0138	0.0173	0.0128	0.0131	0.0121	0.0103	0.0100	0.0097	0.0104	0.0108	0.0103	0.0097	0.0080	0.0083	0.0083	0.0079	0.0073	0.0076	0.0072
WI	0.0118	0.0120	0.0161	0.0124	0.0116	0.0103	0.0091	0.0098	0.0092	0.0091	0.0088	0.0084	0.0082	0.0074	0.0072	0.0065	0.0050	0.0044	0.0044	0.0039
WY	0.0122	0.0139	0.0164	0.0112	0.0110	0.0108	0.0105	0.0111	0.0099	0.0103	0.0120	0.0102	0.0087	0.0087	0.0087	0.0086	0.0067	0.0053	0.0065	0.0072
**Race/ Eth.**																				
AIAN	0.0424	IMP	0.0486	0.0384	0.0404	0.0326	0.0363	0.0391	0.0254	0.0212	0.0288	0.0255	0.0286	0.0195	0.0283	0.0153	0.0188	0.0182	0.0162	0.0193
Asian	0.0123	IMP	0.01	0.0108	0.0127	0.0076	0.0116	0.0099	0.0116	0.0071	0.0104	0.0094	0.0061	0.0071	0.0055	0.0048	0.0052	0.006	0.0055	IMP
Black	0.0053	IMP	0.0055	0.0059	0.0053	0.0062	0.005	0.0036	0.0049	0.0049	0.0048	0.0051	0.0046	0.0039	0.0031	0.0043	0.0033	0.0029	0.0027	0.0024
Latinx	0.0062	IMP	0.0067	0.0065	0.0067	0.0076	0.0059	0.0058	0.0064	0.006	0.0058	0.0054	0.0043	0.0037	0.0042	0.0032	0.003	0.0031	0.0028	0.0031
White	0.0037	IMP	0.0039	0.004	0.0041	0.0036	0.0035	0.003	0.0031	0.0035	0.0035	0.0033	0.0031	0.0031	0.0031	0.0031	0.0027	0.0026	0.0026	0.0023

Notes: IMP= Imputed (see eTable 1).

© 2022 Anderson NW et al. *JAMA Network Open.*

eTable 3. Marijuana Component Data Standard Errors by Year

	‘00	‘01	‘02	‘03	‘04	‘05	‘06	‘07	‘08	‘09	‘10	‘11	‘12	‘13	‘14	‘15	‘16	‘17	‘18	‘19
**USA**	IMP	IMP	IMP	0.0022	0.0021	0.0021	0.0021	0.0022	0.0021	0.0022	0.0023	0.0024	0.0021	0.0021	0.0022	0.0023	0.0022	0.0022	0.0023	0.0024
**State**																				
AL	IMP	IMP	IMP	0.0102	0.0107	0.0108	0.0091	0.0092	0.0090	0.0089	0.0093	0.0090	0.0091	0.0099	0.0092	0.0091	0.0097	0.0092	0.0099	0.0094
AK	IMP	IMP	IMP	0.0134	0.0139	0.0142	0.0119	0.0118	0.0119	0.0126	0.0133	0.0127	0.0132	0.0132	0.0130	0.0140	0.0149	0.0129	0.0133	0.0135
AZ	IMP	IMP	IMP	0.0134	0.0130	0.0137	0.0115	0.0104	0.0111	0.0122	0.0121	0.0123	0.0119	0.0123	0.0120	0.0112	0.0106	0.0101	0.0116	0.0118
AR	IMP	IMP	IMP	0.0119	0.0119	0.0114	0.0108	0.0103	0.0098	0.0103	0.0104	0.0096	0.0099	0.0111	0.0110	0.0104	0.0097	0.0097	0.0099	0.0100
CA	IMP	IMP	IMP	0.0068	0.0070	0.0068	0.0065	0.0064	0.0067	0.0072	0.0074	0.0074	0.0074	0.0072	0.0073	0.0071	0.0068	0.0069	0.0070	0.0076
CO	IMP	IMP	IMP	0.0142	0.0140	0.0128	0.0119	0.0125	0.0123	0.0133	0.0145	0.0139	0.0131	0.0148	0.0151	0.0136	0.0134	0.0139	0.0150	0.0147
CT	IMP	IMP	IMP	0.0134	0.0136	0.0140	0.0114	0.0108	0.0111	0.0129	0.0131	0.0124	0.0128	0.0131	0.0124	0.0128	0.0122	0.0126	0.0135	0.0122
DE	IMP	IMP	IMP	0.0130	0.0126	0.0115	0.0106	0.0103	0.0117	0.0121	0.0129	0.0122	0.0125	0.0130	0.0120	0.0107	0.0123	0.0118	0.0122	0.0123
FL	IMP	IMP	IMP	0.0073	0.0067	0.0063	0.0062	0.0061	0.0066	0.0068	0.0066	0.0062	0.0064	0.0069	0.0072	0.0074	0.0072	0.0076	0.0072	0.0071
GA	IMP	IMP	IMP	0.0115	0.0112	0.0109	0.0097	0.0095	0.0100	0.0107	0.0109	0.0099	0.0103	0.0106	0.0092	0.0085	0.0085	0.0087	0.0094	0.0089
HI	IMP	IMP	IMP	0.0148	0.0142	0.0137	0.0119	0.0121	0.0120	0.0137	0.0125	0.0123	0.0131	0.0128	0.0117	0.0119	0.0121	0.0104	0.0104	0.0109
ID	IMP	IMP	IMP	0.0119	0.0115	0.0108	0.0096	0.0097	0.0097	0.0108	0.0111	0.0108	0.0103	0.0110	0.0116	0.0112	0.0103	0.0107	0.0106	0.0107
IL	IMP	IMP	IMP	0.0065	0.0066	0.0064	0.0061	0.0058	0.0057	0.0064	0.0064	0.0064	0.0062	0.0064	0.0073	0.0076	0.0076	0.0084	0.0090	0.0094
IN	IMP	IMP	IMP	0.0111	0.0113	0.0109	0.0091	0.0100	0.0105	0.0106	0.0099	0.0092	0.0099	0.0103	0.0106	0.0113	0.0113	0.0115	0.0115	0.0106
IA	IMP	IMP	IMP	0.0111	0.0105	0.0103	0.0093	0.0091	0.0087	0.0101	0.0111	0.0100	0.0099	0.0102	0.0097	0.0093	0.0095	0.0098	0.0102	0.0107
KS	IMP	IMP	IMP	0.0110	0.0117	0.0118	0.0102	0.0099	0.0099	0.0104	0.0106	0.0109	0.0099	0.0092	0.0099	0.0103	0.0101	0.0106	0.0105	0.0105
KY	IMP	IMP	IMP	0.0115	0.0122	0.0105	0.0092	0.0109	0.0102	0.0102	0.0101	0.0100	0.0095	0.0093	0.0095	0.0099	0.0101	0.0103	0.0104	0.0104
LA	IMP	IMP	IMP	0.0103	0.0106	0.0106	0.0093	0.0098	0.0095	0.0097	0.0094	0.0084	0.0085	0.0105	0.0102	0.0090	0.0096	0.0092	0.0091	0.0087
ME	IMP	IMP	IMP	0.0131	0.0134	0.0138	0.0121	0.0120	0.0120	0.0117	0.0122	0.0118	0.0117	0.0123	0.0135	0.0136	0.0128	0.0130	0.0134	0.0149
MD	IMP	IMP	IMP	0.0117	0.0125	0.0121	0.0097	0.0097	0.0104	0.0121	0.0117	0.0107	0.0114	0.0120	0.0125	0.0114	0.0114	0.0119	0.0110	0.0107
MA	IMP	IMP	IMP	0.0141	0.0133	0.0128	0.0117	0.0122	0.0117	0.0126	0.0131	0.0122	0.0119	0.0122	0.0126	0.0123	0.0129	0.0133	0.0139	0.0136
MI	IMP	IMP	IMP	0.0071	0.0069	0.0066	0.0062	0.0060	0.0062	0.0065	0.0065	0.0064	0.0068	0.0069	0.0079	0.0083	0.0088	0.0086	0.0086	0.0086
MN	IMP	IMP	IMP	0.0125	0.0127	0.0119	0.0100	0.0102	0.0101	0.0099	0.0098	0.0104	0.0106	0.0104	0.0105	0.0098	0.0108	0.0107	0.0111	0.0117
MS	IMP	IMP	IMP	0.0102	0.0099	0.0100	0.0086	0.0085	0.0082	0.0093	0.0096	0.0097	0.0093	0.0099	0.0097	0.0091	0.0093	0.0098	0.0101	0.0092
MO	IMP	IMP	IMP	0.0117	0.0126	0.0120	0.0101	0.0102	0.0101	0.0100	0.0104	0.0101	0.0099	0.0115	0.0105	0.0105	0.0108	0.0105	0.0108	0.0102
MT	IMP	IMP	IMP	0.0142	0.0139	0.0140	0.0123	0.0122	0.0117	0.0116	0.0127	0.0131	0.0128	0.0117	0.0110	0.0118	0.0131	0.0127	0.0124	0.0124
NE	IMP	IMP	IMP	0.0126	0.0118	0.0102	0.0089	0.0091	0.0100	0.0096	0.0097	0.0094	0.0104	0.0113	0.0101	0.0095	0.0105	0.0106	0.0114	0.0124
NV	IMP	IMP	IMP	0.0143	0.0134	0.0129	0.0114	0.0120	0.0121	0.0125	0.0129	0.0141	0.0145	0.0129	0.0120	0.0115	0.0130	0.0124	0.0131	0.0139
NH	IMP	IMP	IMP	0.0151	0.0137	0.0118	0.0106	0.0117	0.0129	0.0126	0.0141	0.0134	0.0127	0.0132	0.0133	0.0124	0.0117	0.0121	0.0130	0.0133
NJ	IMP	IMP	IMP	0.0118	0.0113	0.0111	0.0101	0.0101	0.0089	0.0103	0.0112	0.0108	0.0106	0.0103	0.0101	0.0093	0.0086	0.0091	0.0093	0.0093
NM	IMP	IMP	IMP	0.0152	0.0144	0.0141	0.0122	0.0127	0.0121	0.0126	0.0132	0.0131	0.0145	0.0139	0.0127	0.0124	0.0126	0.0130	0.0138	0.0147
NY	IMP	IMP	IMP	0.0071	0.0069	0.0074	0.0064	0.0067	0.0066	0.0070	0.0070	0.0068	0.0067	0.0070	0.0074	0.0076	0.0072	0.0070	0.0074	0.0075
NC	IMP	IMP	IMP	0.0129	0.0114	0.0110	0.0100	0.0094	0.0105	0.0104	0.0102	0.0108	0.0112	0.0113	0.0102	0.0090	0.0085	0.0083	0.0091	0.0103
ND	IMP	IMP	IMP	0.0126	0.0124	0.0103	0.0087	0.0098	0.0091	0.0090	0.0089	0.0100	0.0101	0.0093	0.0098	0.0109	0.0110	0.0096	0.0099	0.0100
OH	IMP	IMP	IMP	0.0067	0.0066	0.0065	0.0060	0.0061	0.0062	0.0061	0.0063	0.0063	0.0064	0.0062	0.0068	0.0071	0.0076	0.0076	0.0078	0.0077
OK	IMP	IMP	IMP	0.0127	0.0126	0.0130	0.0107	0.0105	0.0109	0.0116	0.0117	0.0110	0.0103	0.0096	0.0108	0.0107	0.0101	0.0105	0.0107	0.0106

© 2022 Anderson NW et al. *JAMA Network Open.*

OR	IMP	IMP	IMP	0.0131	0.0129	0.0141	0.0129	0.0120	0.0123	0.0128	0.0131	0.0124	0.0122	0.0138	0.0134	0.0130	0.0141	0.0134	0.0133	0.0138
PA	IMP	IMP	IMP	0.0067	0.0067	0.0066	0.0063	0.0060	0.0065	0.0069	0.0069	0.0065	0.0064	0.0067	0.0071	0.0075	0.0073	0.0073	0.0076	0.0080
RI	IMP	IMP	IMP	0.0143	0.0153	0.0155	0.0128	0.0131	0.0133	0.0125	0.0132	0.0136	0.0144	0.0153	0.0136	0.0134	0.0137	0.0128	0.0128	0.0135
SC	IMP	IMP	IMP	0.0111	0.0112	0.0106	0.0093	0.0094	0.0094	0.0099	0.0099	0.0105	0.0105	0.0113	0.0103	0.0098	0.0102	0.0095	0.0110	0.0106
SD	IMP	IMP	IMP	0.0131	0.0128	0.0120	0.0093	0.0093	0.0099	0.0108	0.0106	0.0103	0.0101	0.0099	0.0095	0.0096	0.0107	0.0108	0.0103	0.0100
TN	IMP	IMP	IMP	0.0107	0.0106	0.0111	0.0100	0.0099	0.0096	0.0092	0.0091	0.0094	0.0096	0.0109	0.0105	0.0099	0.0106	0.0101	0.0102	0.0100
TX	IMP	IMP	IMP	0.0067	0.0066	0.0062	0.0058	0.0057	0.0057	0.0061	0.0063	0.0061	0.0062	0.0061	0.0059	0.0067	0.0066	0.0064	0.0066	0.0069
UT	IMP	IMP	IMP	0.0105	0.0102	0.0104	0.0093	0.0089	0.0089	0.0092	0.0091	0.0094	0.0095	0.0098	0.0098	0.0086	0.0089	0.0094	0.0096	0.0098
VT	IMP	IMP	IMP	0.0152	0.0138	0.0128	0.0126	0.0131	0.0125	0.0125	0.0132	0.0139	0.0143	0.0144	0.0138	0.0126	0.0130	0.0147	0.0156	0.0153
VA	IMP	IMP	IMP	0.0125	0.0112	0.0107	0.0104	0.0100	0.0108	0.0112	0.0105	0.0102	0.0108	0.0110	0.0100	0.0086	0.0085	0.0084	0.0092	0.0082
WA	IMP	IMP	IMP	0.0129	0.0122	0.0121	0.0108	0.0109	0.0111	0.0116	0.0119	0.0127	0.0118	0.0123	0.0135	0.0124	0.0117	0.0125	0.0136	0.0139
WV	IMP	IMP	IMP	0.0128	0.0120	0.0109	0.0100	0.0101	0.0103	0.0102	0.0106	0.0106	0.0101	0.0096	0.0097	0.0105	0.0103	0.0097	0.0107	0.0112
WI	IMP	IMP	IMP	0.0125	0.0129	0.0119	0.0097	0.0104	0.0108	0.0111	0.0110	0.0098	0.0104	0.0115	0.0119	0.0113	0.0121	0.0105	0.0103	0.0106
WY	IMP	IMP	IMP	0.0121	0.0117	0.0122	0.0112	0.0111	0.0110	0.0116	0.0118	0.0105	0.0106	0.0104	0.0105	0.0119	0.0119	0.0112	0.0116	0.0117
**Race/ Eth.**																				
AIAN	IMP	IMP	0.0456	0.0339	IMP	0.0332	IMP	IMP	0.0428	0.0323	0.032	0.0285	0.025	0.0352	0.0428	0.0251	0.0387	0.0442	0.0303	0.0336
Asian	0.0118	IMP	0.0108	0.0115	0.0152	0.0079	0.0133	0.0159	0.01	0.01	0.0127	0.0128	0.0085	0.0126	0.0115	0.0091	0.0122	0.0086	0.0109	0.0099
Black	0.0058	IMP	0.0074	0.0074	0.0074	0.0078	0.0071	0.0069	0.0073	0.0078	0.0077	0.0087	0.0081	0.0083	0.0083	0.0082	0.0086	0.0087	0.0095	0.0087
Latinx	0.0069	IMP	0.0091	0.0081	0.0072	0.0084	0.008	0.0069	0.0076	0.0087	0.0088	0.0077	0.0072	0.0076	0.0074	0.0071	0.0066	0.0073	0.0077	0.0079
White	0.0034	IMP	0.004	0.004	0.004	0.0036	0.0035	0.0038	0.0037	0.0039	0.0041	0.0041	0.004	0.004	0.0045	0.0046	0.0042	0.0044	0.0044	0.0046

Notes: IMP= Imputed (see eTable 1).

© 2022 Anderson NW et al. *JAMA Network Open.*

eTable 4. Obesity Component Data Standard Errors by Year

	‘00	‘01	‘02	‘03	‘04	‘05	‘06	‘07	‘08	‘09	‘10	‘11	‘12	‘13	‘14	‘15	‘16	‘17	‘18	‘19
**USA**	IMP	0.0051	IMP	0.0066	IMP	0.0046	IMP	0.0051	IMP	0.0051	IMP	0.0066	IMP	0.0056	IMP	0.0071	IMP	0.0051	IMP	0.0087
**State**																				
AL	IMP	0.0128	IMP	0.0138	IMP	0.0087	IMP	IMP	IMP	0.0112	IMP	0.0179	IMP	0.0128	IMP	0.0133	IMP	IMP	IMP	0.0133
AK	IMP	IMP	IMP	0.0107	IMP	IMP	IMP	0.0102	IMP	0.0102	IMP	0.0097	IMP	0.0097	IMP	0.0107	IMP	0.0102	IMP	0.0117
AZ	IMP	IMP	IMP	0.0087	IMP	0.0097	IMP	0.0112	IMP	0.0092	IMP	0.0092	IMP	0.0122	IMP	0.0107	IMP	0.0107	IMP	0.0133
AR	IMP	0.0092	IMP	IMP	IMP	0.0092	IMP	0.0112	IMP	0.0122	IMP	0.0102	IMP	0.0107	IMP	0.0097	IMP	0.0199	IMP	0.0107
CA	IMP	IMP	IMP	IMP	IMP	IMP	IMP	IMP	IMP	IMP	IMP	IMP	IMP	IMP	IMP	0.0128	IMP	0.0173	IMP	0.0128
CO	IMP	IMP	IMP	IMP	IMP	0.0133	IMP	IMP	IMP	0.0092	IMP	0.0102	IMP	IMP	IMP	IMP	IMP	0.0097	IMP	0.0092
CT	IMP	IMP	IMP	IMP	IMP	0.0117	IMP	0.0077	IMP	0.0097	IMP	0.0122	IMP	0.0107	IMP	0.0107	IMP	0.0102	IMP	0.0122
DE	IMP	0.0056	IMP	0.0071	IMP	0.0071	IMP	0.0077	IMP	0.0071	IMP	0.0071	IMP	0.0066	IMP	0.0087	IMP	0.0102	IMP	IMP
FL	IMP	0.0036	IMP	0.0077	IMP	0.0051	IMP	0.0066	IMP	0.0051	IMP	0.0056	IMP	0.0056	IMP	0.0056	IMP	0.0066	IMP	0.0071
GA	IMP	IMP	IMP	0.0077	IMP	0.0097	IMP	0.0097	IMP	0.0102	IMP	0.0112	IMP	0.0082	IMP	IMP	IMP	IMP	IMP	0.0087
HI	IMP	IMP	IMP	IMP	IMP	0.0097	IMP	0.0133	IMP	0.0158	IMP	0.0112	IMP	0.0092	IMP	0.0097	IMP	0.0056	IMP	0.0102
ID	IMP	0.0066	IMP	0.0082	IMP	0.0082	IMP	0.0077	IMP	0.0071	IMP	0.0071	IMP	0.0071	IMP	0.0102	IMP	0.0087	IMP	0.0107
IL	IMP	IMP	IMP	0.0107	IMP	IMP	IMP	0.0102	IMP	0.0102	IMP	0.0082	IMP	0.0087	IMP	0.0087	IMP	0.0112	IMP	0.0097
IN	IMP	IMP	IMP	IMP	IMP	0.0122	IMP	0.0092	IMP	0.0112	IMP	0.0082	IMP	IMP	IMP	0.0087	IMP	IMP	IMP	IMP
IA	IMP	IMP	IMP	IMP	IMP	0.0138	IMP	0.0133	IMP	IMP	IMP	0.0143	IMP	IMP	IMP	IMP	IMP	0.0168	IMP	0.0148
KS	IMP	IMP	IMP	IMP	IMP	0.0097	IMP	0.0087	IMP	0.0102	IMP	0.0071	IMP	0.0102	IMP	IMP	IMP	0.0092	IMP	0.0122
KY	IMP	IMP	IMP	0.0128	IMP	0.0071	IMP	0.0082	IMP	0.0128	IMP	0.0122	IMP	0.0117	IMP	0.0107	IMP	0.0143	IMP	0.0143
LA	IMP	IMP	IMP	IMP	IMP	IMP	IMP	0.0148	IMP	0.0128	IMP	0.0122	IMP	0.0128	IMP	IMP	IMP	0.0143	IMP	0.0158
ME	IMP	0.0092	IMP	0.0097	IMP	0.0092	IMP	0.0122	IMP	0.0041	IMP	0.0066	IMP	0.0071	IMP	0.0071	IMP	0.0061	IMP	0.0087
MD	IMP	IMP	IMP	IMP	IMP	0.0107	IMP	0.0112	IMP	0.0117	IMP	0.0082	IMP	0.0020	IMP	0.0020	IMP	0.0026	IMP	0.0036
MA	IMP	0.0051	IMP	0.0092	IMP	0.0097	IMP	0.0071	IMP	0.0087	IMP	0.0082	IMP	0.0087	IMP	0.0082	IMP	0.0092	IMP	0.0138
MI	IMP	0.0066	IMP	0.0087	IMP	0.0107	IMP	0.0097	IMP	0.0071	IMP	0.0077	IMP	0.0082	IMP	0.0087	IMP	0.0194	IMP	0.0117
MN	IMP	IMP	IMP	IMP	IMP	IMP	IMP	IMP	IMP	IMP	IMP	IMP	IMP	IMP	IMP	IMP	IMP	IMP	IMP	IMP
MS	IMP	0.0061	IMP	0.0128	IMP	IMP	IMP	0.0112	IMP	0.0128	IMP	0.0107	IMP	0.0117	IMP	0.0097	IMP	IMP	IMP	0.0128
MO	IMP	0.0122	IMP	0.0143	IMP	0.0122	IMP	0.0138	IMP	0.0102	IMP	IMP	IMP	0.0133	IMP	0.0163	IMP	0.0143	IMP	0.0173
MT	IMP	0.0046	IMP	0.0071	IMP	0.0061	IMP	0.0056	IMP	0.0102	IMP	0.0051	IMP	0.0051	IMP	0.0056	IMP	0.0066	IMP	0.0071
NE	IMP	IMP	IMP	0.0071	IMP	0.0056	IMP	IMP	IMP	IMP	IMP	0.0056	IMP	0.0092	IMP	0.0097	IMP	0.0117	IMP	0.0117
NV	IMP	IMP	IMP	IMP	IMP	IMP	IMP	0.0102	IMP	0.0087	IMP	IMP	IMP	0.0092	IMP	0.0097	IMP	0.0107	IMP	0.0102
NH	IMP	IMP	IMP	0.0107	IMP	0.0102	IMP	0.0097	IMP	0.0122	IMP	0.0082	IMP	0.0077	IMP	0.0036	IMP	0.0046	IMP	0.0036
NJ	IMP	0.0082	IMP	IMP	IMP	0.0138	IMP	IMP	IMP	0.0092	IMP	0.0092	IMP	0.0097	IMP	IMP	IMP	IMP	IMP	0.0102
NM	IMP	IMP	IMP	IMP	IMP	0.0117	IMP	0.0097	IMP	0.0122	IMP	0.0097	IMP	0.0112	IMP	0.0041	IMP	0.0082	IMP	0.0087
NY	IMP	IMP	IMP	0.0071	IMP	0.0066	IMP	0.0056	IMP	0.0082	IMP	0.0061	IMP	0.0051	IMP	0.0077	IMP	0.0087	IMP	0.0082
NC	IMP	0.0066	IMP	0.0092	IMP	0.0117	IMP	0.0112	IMP	0.0112	IMP	0.0143	IMP	0.0087	IMP	0.0133	IMP	0.0107	IMP	0.0143
ND	IMP	0.0077	IMP	0.0082	IMP	0.0112	IMP	0.0092	IMP	0.0071	IMP	0.0082	IMP	0.0087	IMP	0.0077	IMP	0.0087	IMP	0.0112
OH	IMP	IMP	IMP	0.0102	IMP	0.0128	IMP	0.0102	IMP	IMP	IMP	0.0143	IMP	0.0112	IMP	IMP	IMP	IMP	IMP	0.0158
OK	IMP	IMP	IMP	0.0092	IMP	0.0097	IMP	0.0092	IMP	0.0138	IMP	0.0143	IMP	0.0092	IMP	0.0148	IMP	0.0138	IMP	0.0097

© 2022 Anderson NW et al. *JAMA Network Open.*

OR	IMP	IMP	IMP	IMP	IMP	IMP	IMP	IMP	IMP	IMP	IMP	IMP	IMP	IMP	IMP	IMP	IMP	IMP	IMP	IMP
PA	IMP	IMP	IMP	IMP	IMP	IMP	IMP	IMP	IMP	0.0071	IMP	IMP	IMP	IMP	IMP	0.0087	IMP	0.0092	IMP	0.0087
RI	IMP	0.0087	IMP	0.0128	IMP	0.0087	IMP	0.0102	IMP	0.0092	IMP	0.0102	IMP	0.0061	IMP	0.0112	IMP	0.0133	IMP	0.0199
SC	IMP	IMP	IMP	IMP	IMP	0.0138	IMP	0.0133	IMP	0.0204	IMP	0.0138	IMP	0.0117	IMP	0.0092	IMP	0.0153	IMP	0.0179
SD	IMP	0.0071	IMP	0.0107	IMP	0.0102	IMP	0.0112	IMP	0.0092	IMP	0.0092	IMP	0.0107	IMP	0.0128	IMP	IMP	IMP	0.0087
TN	IMP	IMP	IMP	0.0102	IMP	0.0128	IMP	0.0092	IMP	0.0097	IMP	0.0077	IMP	0.0092	IMP	0.0097	IMP	0.0128	IMP	0.0148
TX	IMP	0.0097	IMP	IMP	IMP	0.0082	IMP	0.0097	IMP	0.0082	IMP	0.0097	IMP	0.0092	IMP	IMP	IMP	0.0117	IMP	0.0112
UT	IMP	0.0077	IMP	0.0112	IMP	0.0077	IMP	0.0153	IMP	0.0082	IMP	0.0077	IMP	0.0082	IMP	IMP	IMP	0.0082	IMP	0.0087
VT	IMP	0.0092	IMP	0.0092	IMP	0.0107	IMP	0.0143	IMP	0.0071	IMP	0.0092	IMP	0.0097	IMP	0.0026	IMP	0.0020	IMP	0.0026
VA	IMP	IMP	IMP	IMP	IMP	IMP	IMP	IMP	IMP	IMP	IMP	0.0117	IMP	0.0061	IMP	0.0087	IMP	0.0082	IMP	0.0112
WA	IMP	IMP	IMP	IMP	IMP	IMP	IMP	IMP	IMP	IMP	IMP	0.0112	IMP	IMP	IMP	IMP	IMP	IMP	IMP	IMP
WV	IMP	IMP	IMP	0.0158	IMP	0.0112	IMP	0.0112	IMP	0.0112	IMP	IMP	IMP	0.0107	IMP	0.0143	IMP	0.0148	IMP	0.0158
WI	IMP	0.0071	IMP	0.0071	IMP	0.0077	IMP	0.0077	IMP	0.0066	IMP	0.0071	IMP	0.0097	IMP	IMP	IMP	0.0097	IMP	0.0092
WY	IMP	0.0051	IMP	0.0071	IMP	0.0056	IMP	0.0071	IMP	0.0061	IMP	0.0066	IMP	0.0066	IMP	0.0071	IMP	IMP	IMP	IMP
**Race/ Eth.**																				
AIAN	IMP	0.0344	IMP	0.0404	IMP	0.0318	IMP	0.0166	IMP	0.0215	IMP	0.0173	IMP	0.0398	IMP	0.0551	IMP	0.0357	IMP	0.0516
Asian	IMP	0.0193	IMP	0.0194	IMP	0.0092	IMP	0.0147	IMP	0.0116	IMP	0.0207	IMP	0.0134	IMP	0.0118	IMP	0.0166	IMP	0.0164
Black	IMP	0.0122	IMP	0.0072	IMP	0.0083	IMP	0.0112	IMP	0.009	IMP	0.0091	IMP	0.0099	IMP	0.0133	IMP	0.0099	IMP	0.0176
Latinx	IMP	0.0146	IMP	0.0127	IMP	0.0121	IMP	0.0098	IMP	0.008	IMP	0.0081	IMP	0.0109	IMP	0.0084	IMP	0.0064	IMP	0.0128
White	IMP	0.0049	IMP	0.0092	IMP	0.0054	IMP	0.0076	IMP	0.0079	IMP	0.0094	IMP	0.0082	IMP	0.01	IMP	0.0084	IMP	0.0097

Notes: IMP= Imputed (see eTable 1).

© 2022 Anderson NW et al. *JAMA Network Open.*

eTable 5. Fair/Poor Health Component Data Sample Sizes by Year

	'00	'01	'02	'03	'04	'05	'06	'07	'08	'09	'10	'11	'12	'13	'14	'15	'16	'17	'18	'19
**USA**	38,340	71,435	70,721	70,027	68,523	67,420	66,122	65,012	64,147	64,174	64,128	61,517	59,773	59,826	58,245	57,685	53,687	52,830	50,574	49,864
**State**																				
AL	460	1,078	1,097	938	918	875	657	640	627	587	641	596	604	588	626	927	972	1,029	942	919
AK	547	1,296	1,298	1,311	1,133	955	1,024	1,018	959	804	880	852	653	814	723	807	781	725	667	675
AZ	830	1,018	1,026	1,085	1,079	1,020	1,038	997	916	862	859	821	761	792	802	944	1,000	830	902	942
AR	475	765	820	770	806	750	720	732	715	677	572	545	541	719	741	881	1,049	996	936	860
CA	4,257	5,812	5,560	5,571	5,395	5,690	6,375	6,292	6,265	6,113	6,110	5,949	5,837	5,968	5,763	5,741	5,080	5,012	4,752	4,834
CO	605	1,477	1,354	1,413	1,499	1,444	1,437	1,458	1,323	1,387	1,448	1,391	1,371	1,351	1,342	961	657	723	668	614
CT	395	1,234	1,185	1,196	1,289	1,341	1,460	1,404	1,360	1,377	1,374	1,330	1,394	1,236	1,185	871	530	480	426	457
DE	369	852	936	960	824	839	1,018	1,002	893	917	948	967	977	877	850	719	487	491	476	456
FL	1,715	2,666	2,718	2,780	2,591	2,502	2,393	2,316	2,249	2,129	2,219	1,993	2,022	2,095	2,069	2,104	2,068	2,126	1,998	1,923
GA	575	968	906	925	906	1,205	1,373	1,413	1,575	1,497	1,474	1,468	1,365	1,357	1,291	1,369	1,249	1,158	1,208	1,164
HI	372	847	881	821	913	917	965	952	979	991	982	912	902	845	845	819	757	712	644	637
ID	615	1,037	1,116	1,025	965	995	914	955	916	923	931	869	875	818	843	909	965	1,026	918	963
IL	1,656	2,659	2,596	2,460	2,453	2,324	2,025	1,958	2,111	2,112	2,028	1,897	1,996	1,795	1,867	1,638	1,580	1,441	1,428	1,391
IN	476	1,285	1,264	1,388	1,274	1,305	1,120	991	986	1,108	930	1,002	1,006	974	954	985	979	907	913	846
IA	455	1,200	1,223	1,211	1,159	1,104	1,234	1,279	1,213	1,264	1,247	1,185	1,148	1,177	1,144	850	616	623	634	694
KS	469	1,100	1,170	1,256	1,275	1,114	977	895	941	1,046	986	975	921	915	907	809	702	682	699	703
KY	418	915	945	956	919	991	927	888	910	819	908	890	899	735	795	727	646	680	599	545
LA	480	794	792	773	827	667	537	553	590	650	573	559	553	591	665	1,158	1,268	1,211	1,223	1,232
ME	361	996	1,005	982	1,013	1,010	1,059	1,046	1,019	1,027	1,012	950	910	823	734	538	338	388	371	306
MD	361	1,001	1,000	950	962	1,210	1,414	1,454	1,348	1,414	1,402	1,339	1,417	1,412	1,266	941	668	646	678	612
MA	786	1,348	1,316	1,255	1,152	1,087	967	984	876	980	922	942	849	855	813	857	933	991	930	944
MI	1,368	2,020	1,987	2,023	1,976	2,031	1,755	1,712	1,568	1,530	1,482	1,419	1,406	1,313	1,384	1,113	1,029	1,095	1,094	1,073
MN	537	1,417	1,426	1,330	1,416	1,529	1,466	1,503	1,438	1,475	1,565	1,544	1,507	1,528	1,371	980	746	686	713	714
MS	473	711	782	724	703	697	627	599	600	551	628	609	514	534	543	907	1,066	952	940	932
MO	419	1,122	1,088	985	1,019	1,105	1,146	1,054	1,053	1,057	1,137	1,011	977	970	953	804	739	794	702	697
MT	501	791	778	730	673	626	621	626	560	521	540	468	532	512	550	772	978	1,003	858	831
NE	496	1,151	1,199	1,241	1,227	1,142	992	905	950	987	1,030	1,005	1,110	921	887	868	819	736	729	694
NV	671	1,394	1,381	1,455	1,401	1,065	1,047	1,025	1,020	937	982	1,006	888	962	910	860	672	714	661	672
NH	369	1,207	1,000	1,242	1,218	1,150	1,313	1,226	1,306	1,243	1,209	1,100	1,057	1,186	1,006	770	587	575	578	530
NJ	1,061	1,750	1,739	1,736	1,747	1,515	1,358	1,371	1,350	1,441	1,384	1,187	1,171	1,126	1,056	1,084	1,083	1,008	958	906
NM	723	973	915	812	852	747	650	727	593	673	659	587	529	489	539	1,066	1,188	1,075	967	856
NY	2,423	3,524	3,498	3,374	3,207	2,890	2,788	2,667	2,676	2,676	2,627	2,437	2,275	2,243	2,294	2,143	1,881	1,856	1,714	1,839
NC	875	1,331	1,322	1,312	1,301	1,382	1,291	1,197	1,264	1,244	1,243	1,140	1,210	1,134	1,179	1,305	1,281	1,199	1,165	1,184
ND	391	984	1,062	890	853	787	842	786	787	746	773	778	683	734	697	792	709	755	780	726
OH	1,287	2,256	2,193	2,061	1,997	1,966	1,803	1,830	1,712	1,668	1,739	1,694	1,442	1,536	1,466	1,533	1,434	1,428	1,201	1,148
OK	483	987	920	862	733	768	811	852	828	784	747	809	764	797	711	964	860	833	909	850

© 2022 Anderson NW et al. *JAMA Network Open.*

OR	436	1,074	1,108	1,098	953	956	861	890	802	927	867	781	734	773	857	797	764	798	727	753
PA	1,326	2,371	2,321	2,342	2,367	2,124	1,772	1,716	1,864	1,822	1,732	1,614	1,696	1,668	1,543	1,533	1,310	1,302	1,272	1,286
RI	360	1,092	1,085	1,078	1,232	1,198	1,092	994	990	1,028	1,023	952	959	956	797	531	354	370	368	378
SC	360	814	682	814	821	729	829	786	806	793	801	704	602	680	654	791	798	796	751	727
SD	478	1,192	1,205	1,259	1,042	1,050	1,012	1,012	1,005	1,016	1,077	979	1,006	982	940	764	626	576	581	663
TN	405	827	870	866	796	853	901	843	826	772	811	771	782	761	798	875	910	901	834	967
TX	2,540	3,479	3,499	3,514	3,511	3,744	3,872	3,827	3,915	4,073	4,120	4,081	3,805	3,923	3,959	3,844	3,609	3,607	3,521	3,166
UT	716	1,301	1,369	1,351	1,297	1,352	1,113	1,100	1,079	1,051	1,068	1,022	1,032	1,139	1,141	1,145	1,226	1,175	1,112	1,224
VT	341	1,069	1,038	963	950	848	835	809	745	791	740	719	753	707	596	623	588	550	530	516
VA	515	1,140	1,084	1,093	1,127	1,180	1,278	1,360	1,325	1,328	1,327	1,278	1,296	1,292	1,234	1,183	1,027	1,013	965	946
WA	458	1,197	1,220	1,176	1,150	1,233	1,103	1,062	1,069	1,121	1,104	1,127	969	1,079	1,035	1,034	962	971	988	979
WV	369	892	840	911	821	693	601	705	650	606	633	617	566	553	591	806	1,016	992	923	942
WI	545	1,418	1,483	1,390	1,391	1,330	1,308	1,225	1,184	1,245	1,199	1,138	1,080	1,139	1,011	920	677	707	710	671
WY	490	1,095	955	900	917	799	803	810	838	826	874	913	870	838	768	770	815	879	755	738
**Race/ Eth.**																				
AIAN	639	1340	1478	985	931	908	793	743	694	750	777	652	647	728	654	823	817	715	696	662
Asian	1,332	2,878	2,835	2,055	2,300	2,167	2,119	2,237	2,313	2,416	2,553	2,492	2,630	2,571	2,584	2,604	2,382	2,458	2,450	2,463
Black	4,329	8,312	8,211	7,826	7,558	7,182	7,179	7,106	7,072	6,950	7,153	6,587	6,498	6,456	6,154	6,242	5,920	5,541	5,284	5,030
Latinx	8,756	10,495	10,806	12,311	12,492	12,575	13,200	12,967	13,222	13,250	13,644	13,535	13,113	13,391	13,556	13,527	12,794	12,657	12,073	11,843
White	22,959	47,684	46,558	44,594	42,911	42,036	40,242	39,495	38,496	38,260	37,489	35,731	34,465	34,228	32,922	32,065	29,638	29,254	27,884	27,775

© 2022 Anderson NW et al. *JAMA Network Open.*

eTable 6. Preschool Component Data Sample Sizes by Year

	'00	'01	'02	'03	'04	'05	'06	'07	'08	'09	'10	'11	'12	'13	'14	'15	'16	'17	'18	'19
**USA**	388,776	30,639	27,466	30,753	30,808	72,858	72,339	72,970	72,634	73,224	74,520	73,175	71,558	70,338	67,993	66,299	66,343	67,485	67,180	66,750
**State**																				
AL	6,019	399	385	377	370	1,101	1,058	1,127	1,014	1,128	1,144	1,077	1,034	1,032	933	932	951	928	947	954
AK	1,059	278	245	289	254	173	191	185	176	186	196	227	237	239	222	210	209	185	176	207
AZ	7,815	458	447	542	570	1,611	1,602	1,714	1,583	1,551	1,660	1,654	1,633	1,630	1,552	1,558	1,533	1,530	1,478	1,519
AR	3,769	231	222	269	214	633	670	715	724	715	708	737	645	634	653	645	644	624	700	672
CA	51,494	2,981	2,695	3,022	3,085	8,941	8,883	8,818	8,634	9,044	9,411	9,118	9,061	8,764	8,384	7,926	8,139	8,013	7,934	7,844
CO	5,989	438	420	476	473	1,218	1,283	1,238	1,258	1,259	1,268	1,192	1,210	1,180	1,185	1,180	1,179	1,170	1,218	1,192
CT	4,753	363	349	330	314	867	835	801	772	815	798	778	724	741	684	632	618	686	642	595
DE	1,030	233	226	239	252	203	187	192	179	177	196	190	183	182	186	175	151	168	159	179
FL	19,186	1,227	1,161	1,294	1,368	3,747	3,885	3,778	3,849	3,727	3,965	3,856	3,768	3,553	3,467	3,607	3,326	3,578	3,526	3,439
GA	11,884	775	697	892	832	2,381	2,395	2,433	2,533	2,517	2,481	2,358	2,408	2,285	2,283	2,092	2,118	2,177	2,235	2,206
HI	1,530	296	248	231	255	324	306	264	330	326	300	333	347	339	323	311	299	292	307	273
ID	1,969	291	254	308	312	445	437	461	431	480	453	475	424	443	414	390	420	441	430	449
IL	17,631	1,260	1,150	1,264	1,214	3,210	3,216	3,275	3,171	3,075	3,017	2,985	2,847	2,865	2,760	2,769	2,573	2,647	2,727	2,522
IN	8,561	668	603	604	677	1,691	1,606	1,697	1,653	1,684	1,659	1,600	1,528	1,536	1,432	1,409	1,501	1,454	1,498	1,504
IA	3,704	542	427	493	481	746	763	747	751	734	822	760	701	725	698	677	715	722	710	687
KS	3,713	420	365	425	414	704	731	746	726	696	710	763	732	725	654	689	687	718	715	723
KY	5,348	629	571	636	636	997	921	1,028	1,026	1,062	1,050	997	1,049	1,005	1,005	987	1,023	1,027	1,018	995
LA	6,294	589	560	566	576	1,140	1,004	1,031	1,057	1,069	1,107	1,115	1,025	1,035	999	968	888	1,011	948	969
ME	1,453	191	158	206	192	228	251	224	264	245	242	278	248	210	210	217	235	226	204	251
MD	7,155	609	545	592	600	1,356	1,356	1,337	1,361	1,276	1,403	1,307	1,324	1,353	1,288	1,285	1,288	1,238	1,202	1,298
MA	8,018	642	530	641	562	1,486	1,408	1,513	1,449	1,395	1,441	1,413	1,409	1,352	1,340	1,240	1,270	1,257	1,230	1,265
MI	13,346	1,078	919	1,077	1,064	2,443	2,427	2,271	2,313	2,286	2,154	2,118	2,060	2,083	1,984	1,927	1,958	1,999	2,043	1,996
MN	6,597	531	525	526	557	1,243	1,261	1,303	1,321	1,321	1,263	1,294	1,246	1,238	1,234	1,159	1,195	1,229	1,190	1,254
MS	4,279	569	462	575	530	792	699	698	702	674	753	738	707	708	617	656	608	625	621	581
MO	7,482	547	497	619	570	1,501	1,454	1,409	1,505	1,444	1,468	1,420	1,447	1,396	1,360	1,278	1,308	1,388	1,325	1,384
MT	1,164	219	211	238	218	187	188	197	224	177	238	246	217	226	201	176	247	236	213	217
NE	2,293	436	324	357	341	442	456	456	457	487	440	418	460	465	464	432	444	495	473	498
NV	3,074	251	208	249	241	610	622	700	638	650	739	644	681	632	654	597	584	648	608	639
NH	1,532	240	217	260	231	269	268	287	291	310	246	269	229	260	252	201	224	232	221	249
NJ	11,688	892	731	818	801	2,172	2,104	2,106	2,137	1,999	2,129	2,096	1,948	1,985	1,853	1,842	1,736	1,745	1,742	1,739
NM	2,627	244	224	206	241	454	480	448	459	463	469	519	494	483	422	410	425	383	380	344
NY	25,885	1,554	1,291	1,574	1,594	4,139	4,271	4,249	4,062	4,065	4,193	4,310	4,193	4,132	4,030	4,059	3,895	4,003	3,873	3,901
NC	10,531	798	734	788	797	2,230	2,075	2,147	2,205	2,255	2,354	2,152	2,190	2,085	2,039	1,918	2,048	2,002	2,082	2,064
ND	748	242	213	214	205	132	130	117	149	154	157	152	176	173	169	186	178	205	187	210
OH	15,575	1,136	1,001	1,175	1,114	2,949	2,832	2,811	2,780	2,719	2,839	2,797	2,611	2,521	2,534	2,516	2,383	2,511	2,503	2,452
OK	4,707	300	315	323	343	897	835	935	886	952	948	911	935	946	916	887	867	848	900	910

© 2022 Anderson NW et al. *JAMA Network Open.*

OR	4,359	351	302	278	332	854	798	839	850	854	866	846	862	825	828	814	767	818	869	773
PA	15,170	1,131	1,057	1,066	1,155	2,699	2,654	2,742	2,604	2,736	2,672	2,722	2,592	2,591	2,396	2,359	2,493	2,464	2,414	2,350
RI	1,324	234	235	261	249	230	232	238	213	254	201	218	194	205	209	181	162	198	196	176
SC	5,140	340	331	359	392	1,041	1,020	1,035	1,067	1,136	1,104	1,120	1,039	986	948	956	918	990	1,032	1,018
SD	1,042	388	290	395	390	189	213	198	209	212	212	213	234	231	200	230	241	220	237	234
TN	7,236	524	429	520	508	1,524	1,451	1,434	1,537	1,518	1,462	1,463	1,438	1,402	1,364	1,349	1,343	1,364	1,396	1,379
TX	32,038	1,847	1,679	1,965	2,060	6,321	6,555	6,616	6,602	6,916	6,930	6,674	6,544	6,525	6,363	6,227	6,174	6,355	6,322	6,372
UT	4,033	447	395	457	444	937	938	973	953	979	997	959	959	998	951	873	992	943	889	969
VT	714	228	207	222	189	119	135	119	106	143	116	124	124	107	116	110	105	119	119	102
VA	9,059	699	670	700	747	1,820	1,862	1,843	1,886	1,804	1,941	1,871	1,874	1,813	1,810	1,724	1,824	1,812	1,830	1,725
WA	8,182	552	537	545	579	1,566	1,513	1,510	1,580	1,586	1,609	1,661	1,646	1,593	1,626	1,658	1,649	1,741	1,711	1,677
WV	2,146	370	321	349	289	336	340	396	363	366	403	377	363	343	352	306	341	347	324	299
WI	7,095	584	538	556	599	1,326	1,318	1,359	1,343	1,354	1,314	1,339	1,306	1,292	1,139	1,120	1,192	1,194	1,237	1,230
WY	655	260	219	229	229	121	123	129	148	140	161	170	128	164	165	134	130	165	130	136
**Race / Eth.**																				
AIA	4,157	265	230	279	285	585	653	654	600	615	677	998	991	956	957	832	873	856	753	728
Asia	12,580	1,033	1,034	1,117	1,274	3,121	3,184	3,319	3,230	3,305	3,409	3,077	3,223	3,142	3,256	3,148	3,246	3,455	3,289	3,282
Blac	50,299	3,050	2,855	3,272	3,154	7,599	7,556	7,575	7,547	7,694	8,276	8,448	8,005	7,562	7,374	6,904	6,472	6,472	6,149	5,709
Latin	71,026	4,362	4,072	4,801	4,955	13,780	14,224	14,346	14,497	15,555	16,431	16,270	16,099	15,316	14,461	14,304	13,971	13,996	13,968	13,339
Whit	236,944	20,708	18,219	20,154	19,993	45,060	44,033	44,201	43,512	42,654	42,186	40,578	39,471	39,566	38,128	37,285	37,912	38,529	38,906	39,479

© 2022 Anderson NW et al. *JAMA Network Open.*

eTable 7. High School Graduation Component Data Sample Sizes by Year

	'00	'01	'02	'03	'04	'05	'06	'07	'08	'09	'10	'11	'12	'13	'14	'15	'16	'17	'18	'19
**USA**	870,339	870,339	870,339	154,164	154,122	232,885	329,971	429,779	453,111	457,604	460,963	491,718	510,958	526,088	513,582	507,336	501,429	500,522	500,873	500,849
**State**																				
AL	14,014	14,014	14,014	2,070	2,026	3,491	5,128	6,740	7,013	7,091	7,289	7,753	7,963	8,056	7,812	7,716	7,589	7,492	7,524	7,568
AK	2,638	2,638	2,638	1,294	1,338	1,275	1,173	1,012	1,065	1,069	1,148	1,243	1,299	1,288	1,202	1,131	1,109	1,051	1,060	1,001
AZ	15,921	15,921	15,921	2,598	2,612	4,436	6,397	8,571	8,895	9,057	9,189	9,928	10,341	10,505	10,230	10,126	10,248	10,385	10,556	10,579
AR	8,369	8,369	8,369	1,258	1,270	2,094	3,016	3,952	4,148	4,182	4,248	4,436	4,601	4,780	4,796	4,829	4,870	4,899	4,946	4,859
CA	105,662	105,662	105,662	15,394	15,630	27,235	40,258	53,594	55,846	56,767	58,112	61,422	63,401	64,505	63,200	62,189	61,185	60,407	60,210	59,568
CO	13,492	13,492	13,492	2,233	2,245	3,485	5,009	6,544	6,931	7,073	7,099	7,418	7,662	8,003	7,994	7,958	7,882	8,003	8,002	8,132
CT	8,745	8,745	8,745	1,466	1,512	2,327	3,396	4,625	5,005	5,175	5,233	5,828	6,262	6,557	6,292	6,090	5,993	5,964	6,030	5,930
DE	2,921	2,921	2,921	1,282	1,351	1,309	1,236	1,222	1,285	1,291	1,251	1,325	1,392	1,436	1,411	1,333	1,305	1,258	1,302	1,317
FL	41,620	41,620	41,620	6,718	6,915	11,938	17,721	23,813	24,800	25,196	25,387	26,977	27,535	27,874	27,099	26,886	26,565	26,517	26,390	26,498
GA	25,541	25,541	25,541	3,651	3,657	6,377	9,807	13,247	14,089	14,247	14,283	15,223	15,701	16,163	15,758	15,466	15,367	15,324	15,523	15,642
HI	4,222	4,222	4,222	1,381	1,363	1,465	1,646	1,749	1,840	1,841	1,891	2,046	2,183	2,303	2,297	2,224	2,173	2,065	2,083	2,060
ID	4,882	4,882	4,882	1,621	1,633	1,728	1,889	2,042	2,136	2,178	2,174	2,238	2,224	2,325	2,309	2,280	2,157	2,178	2,248	2,353
IL	36,926	36,926	36,926	5,966	5,979	9,652	13,894	18,214	18,987	19,155	19,216	20,007	20,442	20,744	20,285	19,746	19,206	18,905	18,634	18,222
IN	20,049	20,049	20,049	3,241	3,244	5,039	7,523	9,800	10,461	10,531	10,721	11,326	11,687	11,999	11,940	11,823	11,613	11,495	11,521	11,636
IA	10,381	10,381	10,381	2,585	2,536	2,932	3,616	4,385	4,760	4,759	4,699	5,015	5,279	5,594	5,452	5,405	5,421	5,532	5,575	5,522
KS	9,364	9,364	9,364	2,487	2,480	2,905	3,575	4,081	4,302	4,183	4,172	4,336	4,601	4,793	4,722	4,613	4,609	4,689	4,776	4,812
KY	13,475	13,475	13,475	3,310	3,199	3,950	4,899	6,040	6,248	6,377	6,331	6,786	7,040	7,384	7,338	7,326	7,147	7,155	7,114	7,211
LA	15,880	15,880	15,880	3,314	3,263	4,519	5,486	6,537	6,343	6,425	6,460	6,765	6,935	6,952	6,776	6,543	6,375	6,404	6,386	6,303
ME	3,708	3,708	3,708	950	957	1,194	1,478	1,763	1,866	1,893	1,880	2,066	2,171	2,288	2,137	2,142	2,090	2,158	2,065	2,079
MD	14,368	14,368	14,368	2,786	2,927	4,297	5,968	7,687	8,224	8,343	8,398	9,008	9,418	9,649	9,335	9,188	9,059	8,894	8,725	8,738
MA	18,361	18,361	18,361	2,810	2,779	4,367	6,847	9,323	10,327	10,553	10,650	12,301	13,582	14,661	13,992	13,749	13,847	14,052	14,042	13,865
MI	30,101	30,101	30,101	5,278	5,304	8,054	11,392	14,702	15,419	15,419	15,435	15,843	15,979	16,127	15,669	15,467	15,148	15,044	14,920	14,868
MN	15,533	15,533	15,533	2,838	2,807	4,039	5,646	7,248	7,643	7,638	7,567	7,870	7,986	8,190	7,913	7,816	7,709	7,707	7,699	7,568
MS	11,393	11,393	11,393	3,035	2,857	3,279	3,899	4,571	4,733	4,757	4,755	5,117	5,347	5,551	5,429	5,402	5,186	5,145	5,022	5,068
MO	17,669	17,669	17,669	3,060	2,985	4,636	6,715	8,781	9,149	9,054	9,072	9,763	10,253	10,411	10,024	9,853	10,026	10,130	10,110	9,914
MT	3,190	3,190	3,190	1,296	1,267	1,228	1,233	1,204	1,231	1,198	1,284	1,349	1,433	1,495	1,533	1,503	1,393	1,350	1,322	1,380
NE	6,002	6,002	6,002	1,772	1,652	1,805	2,155	2,415	2,570	2,553	2,670	2,887	2,995	3,152	3,133	3,153	3,161	3,150	3,193	3,142
NV	5,618	5,618	5,618	1,123	1,140	1,726	2,408	3,161	3,377	3,516	3,595	3,681	3,702	3,690	3,679	3,613	3,620	3,608	3,729	3,813
NH	3,741	3,741	3,741	1,205	1,195	1,336	1,544	1,941	2,110	2,141	1,993	2,202	2,358	2,483	2,390	2,393	2,365	2,395	2,408	2,424
NJ	20,716	20,716	20,716	3,402	3,532	5,848	8,717	11,596	12,199	12,069	11,949	12,537	13,023	13,282	12,980	12,787	12,531	12,379	12,378	12,280
NM	5,996	5,996	5,996	1,203	1,175	1,667	2,232	2,741	2,806	2,760	2,782	2,968	3,122	3,247	3,083	2,951	2,787	2,750	2,678	2,716
NY	54,028	54,028	54,028	8,050	8,035	13,543	20,455	27,962	30,006	30,427	30,396	33,058	34,971	36,441	35,214	34,457	33,979	33,243	32,680	32,130
NC	24,792	24,792	24,792	3,651	3,640	5,902	9,152	12,736	13,961	14,173	14,172	15,411	16,174	16,917	16,571	16,229	16,010	16,115	16,514	16,864
ND	3,021	3,021	3,021	1,568	1,574	1,355	1,203	1,079	1,142	1,148	1,122	1,217	1,233	1,303	1,273	1,277	1,259	1,287	1,314	1,281
OH	34,852	34,852	34,852	5,722	5,613	9,008	13,014	17,051	17,705	17,708	17,584	18,776	19,367	20,191	19,603	19,618	19,197	19,182	19,058	19,204
OK	11,556	11,556	11,556	1,744	1,679	2,649	3,794	5,070	5,362	5,463	5,582	5,874	6,162	6,415	6,393	6,418	6,219	6,266	6,325	6,474

© 2022 Anderson NW et al. *JAMA Network Open.*

OR	10,223	10,223	10,223	1,679	1,681	2,659	3,772	4,879	5,104	5,158	5,245	5,632	5,799	5,984	5,819	5,834	5,794	5,863	5,982	6,134
PA	35,879	35,879	35,879	5,661	5,630	9,028	13,745	18,632	20,042	20,146	19,838	21,544	22,680	23,784	22,969	22,751	22,522	22,298	22,103	21,802
RI	4,002	4,002	4,002	1,245	1,292	1,356	1,562	1,744	1,918	1,945	1,940	2,151	2,358	2,490	2,424	2,326	2,274	2,277	2,199	2,221
SC	13,197	13,197	13,197	1,792	1,767	2,984	4,719	6,490	7,030	7,225	7,357	8,079	8,426	8,650	8,343	8,322	8,409	8,520	8,673	8,819
SD	3,441	3,441	3,441	2,058	2,029	1,772	1,454	1,179	1,223	1,197	1,220	1,373	1,469	1,515	1,461	1,452	1,437	1,472	1,446	1,437
TN	17,271	17,271	17,271	2,715	2,718	4,357	6,419	8,490	8,911	8,934	8,974	9,494	9,762	10,133	10,101	10,213	10,070	10,030	10,156	10,381
TX	67,929	67,929	67,929	9,169	9,167	16,489	24,904	33,540	34,879	35,346	36,038	38,251	39,818	40,820	40,285	39,974	39,894	40,456	40,993	41,156
UT	9,824	9,824	9,824	2,147	2,123	2,781	3,490	4,286	4,482	4,582	4,754	4,788	4,794	4,763	4,700	4,862	4,963	5,447	5,736	5,991
VT	2,457	2,457	2,457	1,053	1,091	994	988	919	1,046	1,044	1,112	1,208	1,297	1,312	1,225	1,204	1,207	1,257	1,257	1,282
VA	21,667	21,667	21,667	3,293	3,326	5,383	8,219	11,213	12,162	12,296	12,147	13,069	13,810	14,554	14,378	14,353	14,234	14,081	14,019	13,983
WA	17,885	17,885	17,885	3,005	3,110	4,821	6,937	8,947	9,357	9,506	9,668	10,348	10,687	10,804	10,505	10,390	10,332	10,513	10,562	10,948
WV	6,212	6,212	6,212	1,932	1,824	1,979	2,224	2,504	2,608	2,516	2,529	2,757	2,934	3,021	2,934	2,879	2,836	2,755	2,809	2,796
WI	16,881	16,881	16,881	2,971	2,943	4,363	6,317	8,158	8,531	8,397	8,376	8,849	9,026	9,149	8,874	8,723	8,649	8,553	8,534	8,567
WY	2,336	2,336	2,336	1,319	1,301	1,160	956	755	794	814	831	836	828	804	815	866	906	902	845	872
**Race/ Eth.**																				
AIAN	9,111	9,111	9,111	1,434	1,457	2,177	3,064	3,997	4,238	4,242	4,396	4,940	5,567	5,852	5,621	5,257	5,010	5,023	4,969	4,877
Asian	31,993	31,993	31,993	6,037	6,230	9,554	13,724	18,235	19,793	20,335	20,887	22,943	24,826	26,700	27,062	27,753	28,460	29,066	29,856	30,166
Black	109,582	109,582	109,582	16,018	15,715	24,805	36,778	49,477	53,718	55,434	57,275	63,965	67,993	69,995	66,601	64,099	62,032	60,219	58,764	57,394
Latinx	138,677	138,677	138,677	20,205	20,572	34,951	51,793	69,057	73,510	75,922	79,367	85,471	90,035	92,672	92,284	92,639	93,358	94,715	95,841	96,624
White	559,063	559,063	559,063	106,844	106,625	156,339	217,194	278,985	290,634	289,711	286,290	299,990	306,507	313,347	304,162	299,438	294,155	292,346	291,823	291,828

Notes: Sample sizes shown here are three year estimates assigned to the last year, as used in the analysis. 2000 and 2001 estimates are the same as 2002.

© 2022 Anderson NW et al. *JAMA Network Open.*

eTable 8. Food Security Data Sample Sizes by Year

	‘00	‘01	‘02	‘03	‘04	‘05	‘06	‘07	‘08	‘09	‘10	‘11	‘12	‘13	‘14	‘15	‘16	‘17	‘18	‘19
**USA**	92,222	92,222	92,222	95,826	95,692	92,866	90,769	80,552	76,910	75,755	81,722	80,565	78,338	75,206	74,245	71,258	69,955	65,319	62,513	57,451
**State**																				
AL	1,371	1,371	1,371	1,385	1,280	1,131	942	747	696	724	832	852	814	788	891	1,064	1,207	1,152	1,058	965
AK	1,513	1,513	1,513	1,647	1,582	1,481	1,403	1,272	1,182	1,197	1,234	1,179	1,066	1,005	1,055	1,025	1,061	966	879	770
AZ	1,537	1,537	1,537	1,533	1,536	1,416	1,394	1,178	1,030	932	1,013	1,049	1,040	1,025	1,136	1,230	1,312	1,229	1,176	1,107
AR	1,077	1,077	1,077	1,155	1,160	1,100	1,038	905	862	894	979	947	954	934	1,129	1,230	1,342	1,213	1,090	975
CA	7,376	7,376	7,376	7,159	7,241	7,519	7,685	7,098	6,708	6,694	7,285	7,190	7,016	6,830	6,608	6,239	6,016	5,675	5,602	5,165
CO	1,704	1,704	1,704	1,959	1,983	2,006	2,032	1,863	1,835	1,819	1,932	1,834	1,788	1,717	1,497	1,117	832	730	692	595
CT	1,421	1,421	1,421	1,637	1,606	1,606	1,729	1,586	1,599	1,613	1,798	1,756	1,626	1,505	1,269	949	655	486	484	454
DE	1,109	1,109	1,109	1,151	1,150	1,182	1,281	1,225	1,144	1,091	1,172	1,152	1,144	1,035	981	862	796	680	622	523
FL	3,570	3,570	3,570	3,541	3,399	3,218	3,026	2,690	2,526	2,464	2,637	2,518	2,458	2,377	2,482	2,501	2,559	2,427	2,238	2,033
GA	1,269	1,269	1,269	1,232	1,482	1,679	1,931	1,796	1,782	1,784	1,956	1,841	1,713	1,614	1,697	1,755	1,724	1,626	1,526	1,379
HI	991	991	991	1,190	1,222	1,323	1,345	1,293	1,192	1,132	1,166	1,163	1,178	1,104	1,126	1,022	994	850	834	771
ID	1,468	1,468	1,468	1,430	1,323	1,273	1,360	1,296	1,230	1,115	1,161	1,187	1,208	1,205	1,205	1,133	1,213	1,232	1,277	1,243
IL	3,452	3,452	3,452	3,426	3,377	3,128	2,866	2,454	2,401	2,377	2,537	2,571	2,558	2,494	2,273	2,127	2,030	1,912	1,762	1,621
IN	1,666	1,666	1,666	1,893	1,860	1,741	1,606	1,369	1,274	1,260	1,322	1,279	1,229	1,216	1,224	1,248	1,218	1,102	978	951
IA	1,475	1,475	1,475	1,670	1,696	1,720	1,727	1,545	1,540	1,543	1,626	1,517	1,460	1,424	1,241	999	825	776	816	754
KS	1,515	1,515	1,515	1,725	1,758	1,644	1,514	1,277	1,216	1,205	1,357	1,341	1,371	1,312	1,306	1,128	1,055	904	911	828
KY	1,188	1,188	1,188	1,318	1,265	1,205	1,137	1,018	1,010	1,037	1,183	1,214	1,198	1,093	1,040	975	892	799	750	744
LA	1,107	1,107	1,107	1,081	1,012	903	826	720	745	778	858	842	795	789	1,070	1,326	1,580	1,517	1,461	1,363
ME	1,256	1,256	1,256	1,416	1,469	1,524	1,535	1,407	1,341	1,341	1,372	1,249	1,139	1,025	903	749	630	551	502	443
MD	1,349	1,349	1,349	1,478	1,542	1,701	1,892	1,810	1,738	1,657	1,765	1,729	1,720	1,657	1,537	1,263	993	841	789	721
MA	1,659	1,659	1,659	1,673	1,640	1,494	1,365	1,171	1,158	1,152	1,226	1,152	1,100	1,094	1,100	1,082	1,113	1,060	1,097	1,025
MI	2,929	2,929	2,929	2,843	2,752	2,570	2,394	2,014	1,899	1,754	1,828	1,776	1,754	1,746	1,667	1,527	1,399	1,352	1,354	1,319
MN	1,690	1,690	1,690	1,849	1,987	2,098	2,223	1,991	1,924	1,827	2,007	2,068	2,103	2,076	1,783	1,407	1,045	860	856	852
MS	1,094	1,094	1,094	1,070	960	876	874	835	803	758	783	731	707	678	919	1,089	1,382	1,455	1,489	1,290
MO	1,361	1,361	1,361	1,462	1,516	1,601	1,626	1,414	1,299	1,293	1,373	1,376	1,364	1,346	1,250	1,132	1,060	932	875	815
MT	1,137	1,137	1,137	1,051	987	937	922	827	729	644	693	743	731	680	789	1,028	1,303	1,276	1,153	1,049
NE	1,590	1,590	1,590	1,678	1,604	1,514	1,438	1,267	1,247	1,288	1,348	1,246	1,184	1,110	1,096	1,031	999	895	791	693
NV	1,764	1,764	1,764	1,902	1,890	1,661	1,527	1,295	1,225	1,169	1,262	1,253	1,177	1,082	1,059	1,034	1,041	961	885	790
NH	1,342	1,342	1,342	1,576	1,623	1,707	1,850	1,773	1,623	1,504	1,507	1,493	1,442	1,406	1,260	1,085	925	812	790	743
NJ	2,361	2,361	2,361	2,310	2,285	2,107	1,909	1,589	1,563	1,604	1,716	1,608	1,469	1,367	1,339	1,370	1,365	1,281	1,128	1,045
NM	1,196	1,196	1,196	1,174	1,178	1,062	965	782	702	658	674	657	611	573	785	1,041	1,303	1,326	1,220	1,062
NY	4,574	4,574	4,574	4,548	4,327	3,986	3,656	3,166	2,903	2,902	3,117	3,051	2,768	2,637	2,618	2,519	2,399	2,205	2,122	2,059
NC	1,677	1,677	1,677	1,734	1,870	1,879	1,772	1,524	1,434	1,452	1,561	1,540	1,548	1,505	1,576	1,538	1,539	1,404	1,323	1,236
ND	1,407	1,407	1,407	1,394	1,292	1,286	1,255	1,102	1,024	1,007	1,072	1,062	1,017	944	893	942	1,050	1,067	1,032	957
OH	3,128	3,128	3,128	3,016	3,103	2,872	2,653	2,172	2,163	2,212	2,418	2,344	2,202	2,127	2,117	2,126	2,008	1,832	1,662	1,501
OK	1,234	1,234	1,234	1,151	1,176	1,233	1,312	1,205	1,113	1,043	1,073	1,002	937	912	1,062	1,095	1,153	1,022	967	857

© 2022 Anderson NW et al. *JAMA Network Open.*

OR	1,306	1,306	1,306	1,486	1,401	1,302	1,177	987	1,001	1,103	1,232	1,202	1,114	1,044	1,007	977	996	1,000	949	900
PA	3,249	3,249	3,249	3,260	3,252	2,919	2,569	2,124	2,097	2,057	2,211	2,194	2,207	2,177	2,091	1,933	1,801	1,665	1,560	1,399
RI	1,258	1,258	1,258	1,512	1,510	1,423	1,405	1,283	1,300	1,301	1,398	1,379	1,328	1,198	984	694	564	484	514	465
SC	992	992	992	1,073	1,185	1,214	1,219	996	917	919	1,031	1,040	985	921	925	974	1,008	958	854	763
SD	1,578	1,578	1,578	1,726	1,616	1,478	1,437	1,319	1,256	1,236	1,383	1,418	1,423	1,384	1,262	1,070	926	881	866	768
TN	1,082	1,082	1,082	1,125	1,124	1,121	1,175	1,080	1,055	1,013	1,101	1,063	1,031	991	1,077	1,123	1,190	1,149	1,169	1,167
TX	4,657	4,657	4,657	4,587	4,784	4,872	5,087	4,701	4,454	4,351	4,803	4,895	4,970	4,799	4,814	4,467	4,283	3,926	3,817	3,516
UT	1,751	1,751	1,751	1,927	1,908	1,767	1,625	1,371	1,295	1,299	1,417	1,452	1,428	1,363	1,372	1,456	1,655	1,609	1,559	1,293
VT	1,138	1,138	1,138	1,376	1,294	1,157	1,088	968	984	981	1,023	968	962	1,000	973	883	778	714	646	584
VA	1,264	1,264	1,264	1,344	1,518	1,648	1,793	1,600	1,514	1,417	1,597	1,615	1,660	1,590	1,522	1,389	1,278	1,207	1,174	1,125
WA	1,484	1,484	1,484	1,669	1,780	1,680	1,587	1,311	1,197	1,208	1,469	1,577	1,554	1,397	1,342	1,289	1,290	1,283	1,291	1,232
WV	1,179	1,179	1,179	1,224	1,125	962	884	806	787	781	833	845	798	778	920	1,039	1,203	1,154	1,121	969
WI	1,753	1,753	1,753	2,019	1,975	1,863	1,731	1,522	1,463	1,421	1,516	1,521	1,474	1,450	1,304	1,203	1,049	983	975	935
WY	1,378	1,378	1,378	1,376	1,370	1,293	1,221	1,087	1,071	1,088	1,144	1,132	1,059	980	968	1,032	1,091	1,077	1,042	958
**Race/ Eth.**																				
AIAN	1,814	1,814	1,814	1,679	1,447	1,164	1,069	921	876	936	980	953	886	847	958	1,019	1,080	999	890	786
Asian	3,470	3,470	3,470	3,387	3,132	2,749	2,749	2,577	2,617	2,716	3,072	3,223	3,248	3,176	3,129	3,016	3,080	2,944	2,867	2,538
Black	10,724	10,724	10,724	10,777	10,330	9,825	9,565	8,498	7,987	8,062	8,995	8,910	8,498	7,996	8,067	7,813	7,589	6,884	6,291	5,667
Latinx	12,598	12,598	12,598	13,858	14,874	15,380	15,429	14,043	13,620	13,586	15,140	15,198	15,143	14,662	14,878	14,545	14,413	13,419	12,874	11,827
White	62,628	62,628	62,628	64,310	63,149	60,112	58,224	51,192	48,689	47,424	50,285	49,006	47,298	45,323	43,965	41,750	40,670	38,198	36,796	34,099

Notes: Sample sizes shown here are three year estimates assigned to the last year, as used in the analysis. 2000 and 2001 estimates are the same as 2002.

© 2022 Anderson NW et al. *JAMA Network Open.*

eTable 9. Child and Adolescent Thriving Index 1.0 Weights

Objective Component	Weight	
	Mean (as % of total)	Standard Deviation
Food Security	0.081 (16.6%)	0.018
Enrolled in Preschool	0.010 (2.1%)	0.004
High School Graduation	0.116 (23.8%)	0.015
Proficient Test Score in Math	0.021 (4.3%)	0.008
Proficient Test Score in Reading	0.022 (4.5%)	0.007
Non-Obese	0.008 (1.6%)	0.004
Non-Fair/Poor Health	0.011 (2.3%)	0.009
Non-Low Birthweight	0.021 (4.3%)	0.007
Didn't Smoke Cigarettes in Past Month	0.078 (16.0%)	0.011
Didn't Smoke Marijuana in Past Year	0.028 (5.7%)	0.007
Complement (1 Minus) Juvenile Arrest Rate	0.011 (2.3%)	0.006

Source: Anderson, N.W., Markowitz, A.J., Eisenberg, D. *et al.* The Child and Adolescent Thriving Index 1.0: Developing a Measure of the Outcome Indicators of Well-Being for Population Health Assessment. *Child Ind Res* (2022). https://doi.org/10.1007/s12187-022-09962-0.

Notes: Weight values and standard deviations in this table are calculated based on 10,000 Monte Carlo simulations, and may differ slightly from the point estimates reported in the original paper.

© 2022 Anderson NW et al. *JAMA Network Open.*

eAppendix. Uncertainty Analysis and Monte Carlo Simulation Methodology

Child and adolescent well-being indices may hold less credibility if their results are subject to high levels of uncertainty. This is of particular concern for making the kind of state-level comparisons KIDS COUNT emphasizes. If, for example, the median-ranked state could plausibly lie anywhere between the 20th and 80th percentile, then the ranking is unlikely to be seen as trustworthy enough to motivate real policy change.

There are several types of uncertainties in an index methodology that can be assessed. Nardo and co-authors outline at least seven such sources: [1]

a) selection of sub-indicators: are the right measures chosen in the model selection process?
b) data quality: how accurate are the population-level data that are being used?
c) data editing: in instances where the population-level data are missing, is there an imputation procedure and how much uncertainty is there given the chosen method?
d) data normalization: typically, measures are standardized, but they could instead be ranked or rescaled.
e) weighting scheme: there are three general families: data driven, normative, and hybrid.
f) weights' values: given the weighting scheme, how much uncertainty is there surrounding the values of the weights?
g) composite indicator formula: typically, indices assume an additive nature, but there are other possible specifications (i.e., multiplicative) which assume deficiencies in one domain cannot be simply offset by strengths in another.

Previous analyses of CWI and KIDS COUNT suggest that uncertainty surrounding selection of sub-indicators and within a subset of the weighting schemes is not of great concern. [2-4] However, to this point, the other categories remain largely unexamined. Furthermore, these areas may interact with one another in important ways: one analysis of another composite measure of health, the County Health Rankings, found that uncertainty of rankings when using a data-driven weighting scheme was significantly worse when state-level data quality was poor. [5]

We perform a Monte Carlo analysis with 10,000 simulations, with a focus on two of these categories, as well as their interaction: data quality and weights' values. We report 90% credible intervals (the range of values this percentage of simulations cover), as well as several

© 2022 Anderson NW et al. *JAMA Network Open.*

related metrics, for index values and state rankings. The following parameters are allowed to vary in the simulation:

- *Data Quality*: quality of underlying data can be further divided into two important types: accuracy (how close the average estimate is to the "true value") and precision (how tight is the spread of the estimates). Since the "true value" for component data can not be realistically ascertained, we restrict our analysis of data quality to the latter, which we henceforth call *data variability*. This is done by sampling prevalence rates of underlying component data from a normal distribution using the estimated means and standard errors of the population estimates.
- *Weights' Values*: in the original methodological paper, component weights were constructed as a weighted average of results from several regressions.[6] Using the standard errors of the estimates for each regression, we simulate a distribution of the weights.

A final set of simulations accounts for both data variability and variation in weights, to assess overall uncertainty from these two sources together.

The shaded region in eFigure 1 represents the 90% credible interval from the Monte Carlo simulation for uncertainty from data variability and weights' values. The results suggest two things. First, overall child and adolescent well-being unambiguously improved over the study period: the 90% credible interval for the annual improvement in well-being is narrow (0.003, 0.004 points annually, or between 0.060 and 0.072 points over the 20 year period). Second, there is greater relative uncertainty introduced from the values of the weights, compared to the quality of data. In any given year, variance in the weights' values makes up more than 99% of the total variance when both sources of uncertainty are assessed. This suggests that

© 2022 Anderson NW et al. *JAMA Network Open.*

sample sizes for existing data elements of child and adolescent well-being are sufficiently large, at least at the national level.

Other sources of uncertainty are assessed separately as a robustness check, which are described in more detail below. Across each of these specifications, it is clear that national well-being improved over the study period, which is consistent with existing findings using the KIDS COUNT and CWI indices.

Selection of Sub-Indicators (eFigure 2): In the analysis deriving the Child and Adolescent Thriving Index 1.0, several alternative specifications were considered. For example, the preferred version uses only child-centered components. However, many indices in the literature incorporate contextual measures, such as family poverty or education. Additionally, the model-selection procedure from the methodological paper identifies some alternatives that produce similar results on an individual level but remain untested on a population level.

Weighting Scheme (eFigure 3): We compare the results of Child and Adolescent Thriving Index 1.0, which uses a hybrid weighting scheme known as hedonic weighting, to the KIDS COUNT index, which employs equal-weighting. [7]

Data Normalization (eFigure 4): For our preferred methodology, we opted not to standardize the components before weighting and aggregating because standardized scores are difficult for a lay audience to interpret. Since standardizing is the typical procedure, we conduct a sensitivity with this normalization to investigate the extent to which results change.

Composite Indicator Formula (eFigure 5): Standard aggregation procedure for indices is to simply add the weighted components together. However, this raises concerns of substitutability – situations in which lower values in one area to boost others can drive up the total index score.

© 2022 Anderson NW et al. *JAMA Network Open.*

This property is relevant to the extent that there are occurrences where states can choose to invest public dollars to improve one type of outcome at the expense of others. Therefore, as a robustness check we apply an alternative aggregation methodology that addresses substitutability by using a more multiplicative index, [8] in which component values that are closer to one another result in higher scale scores hold the sum across components constant.

Assessing Uncertainty at the State-Level

State ranking results across the Monte Carlo simulations are presented in eFigure 6 and eTables 10 and 11. In the figure, interquartile ranges for state rankings are shown in the thick bar, and the 90% credible interval is contained in the whiskers. Uncertainty of state rankings is fairly low: When examining both data variability and the weights' values, 86.6% of simulated state rankings are within +-5 positions of the state's actual ranking. These percentages are lower in earlier years (79.7% for 2000-2004), for states ranked near the median (76.0% for states ranked 21-30), and for less populated states (73.3% for the bottom 10 states).

Unlike the national trends, uncertainty in state rankings is largely driven by data variability, rather than the values of the component weights. When looking at uncertainty from only the weights' values, 98.5% of simulated state rankings are within +-5 positions of the state's actual rank. This rate falls to 89.3% when just looking at uncertainty from data variability. Uncertainty in state rankings from other sources in Nardo et al's framework are shown in eFigures 7 to 11.

© 2022 Anderson NW et al. *JAMA Network Open.*

eFigure 1. National Child and Adolescent Thriving Index 1.0, 2000-2019

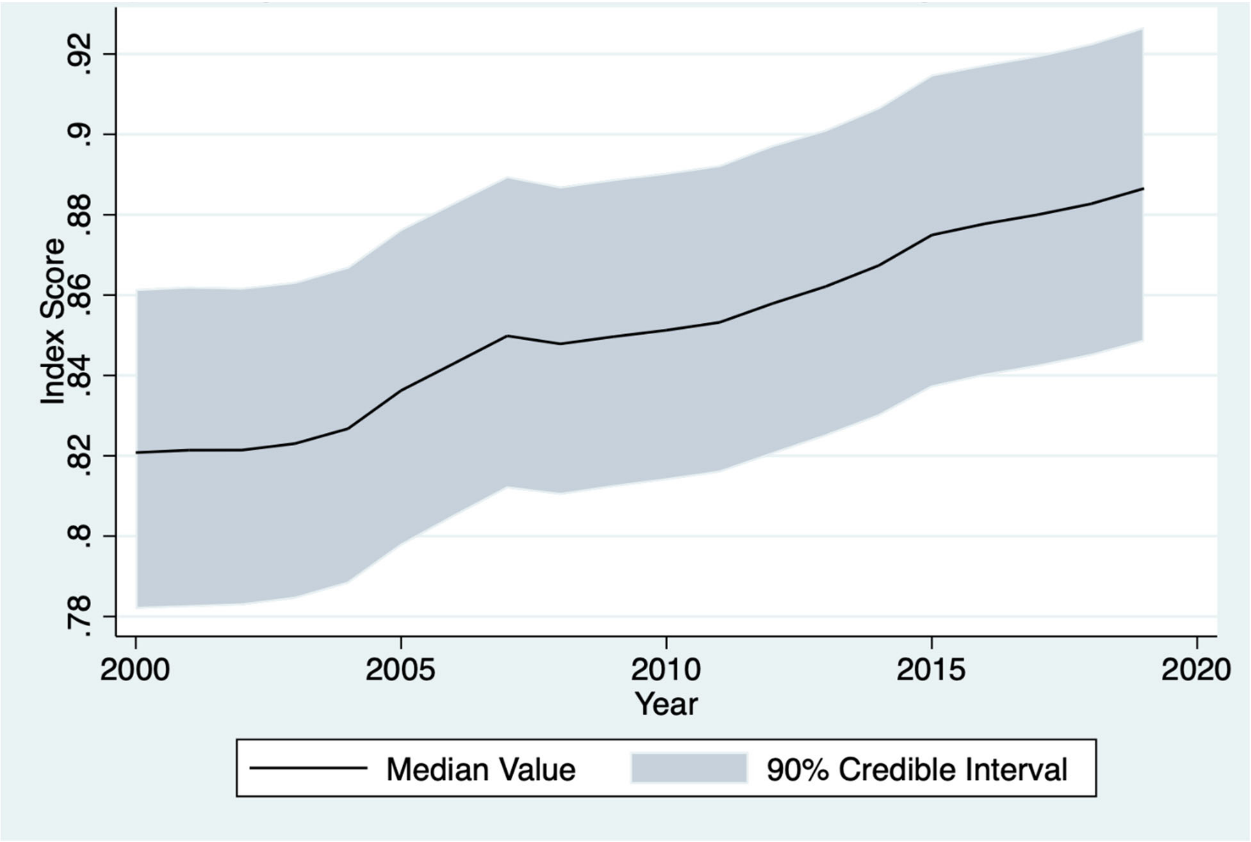

Notes: Credible interval based on 10,000 Monte Carlo simulations for data variability and weights' values.

© 2022 Anderson NW et al. *JAMA Network Open.*

eFigure 2. Robustness of Selection of Indicators Comparing to Alternate Variable Selection Results

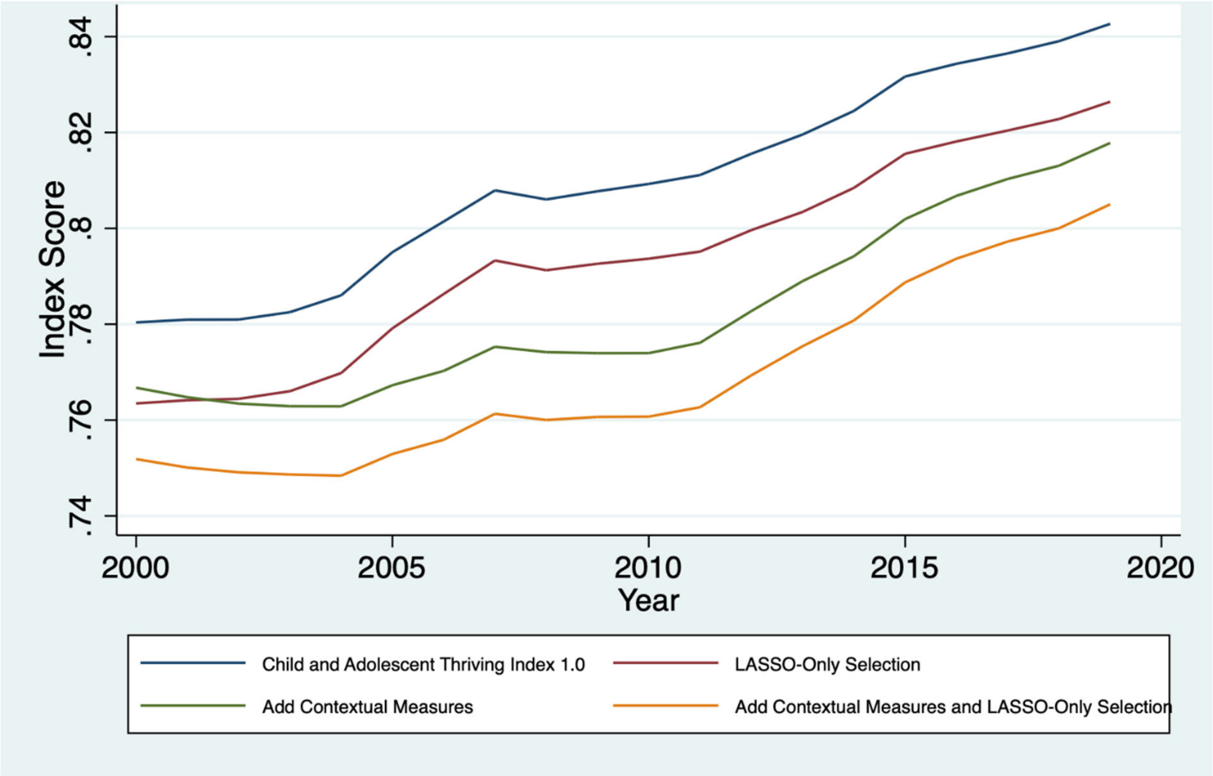

Notes: This graph shows several alternative national-level indices using variations on the model-selection processes from the original methodological paper.

© 2022 Anderson NW et al. *JAMA Network Open.*

eFigure 3. Robustness of Weighting Scheme Comparing to KIDS COUNT (Equally Weighted Components)

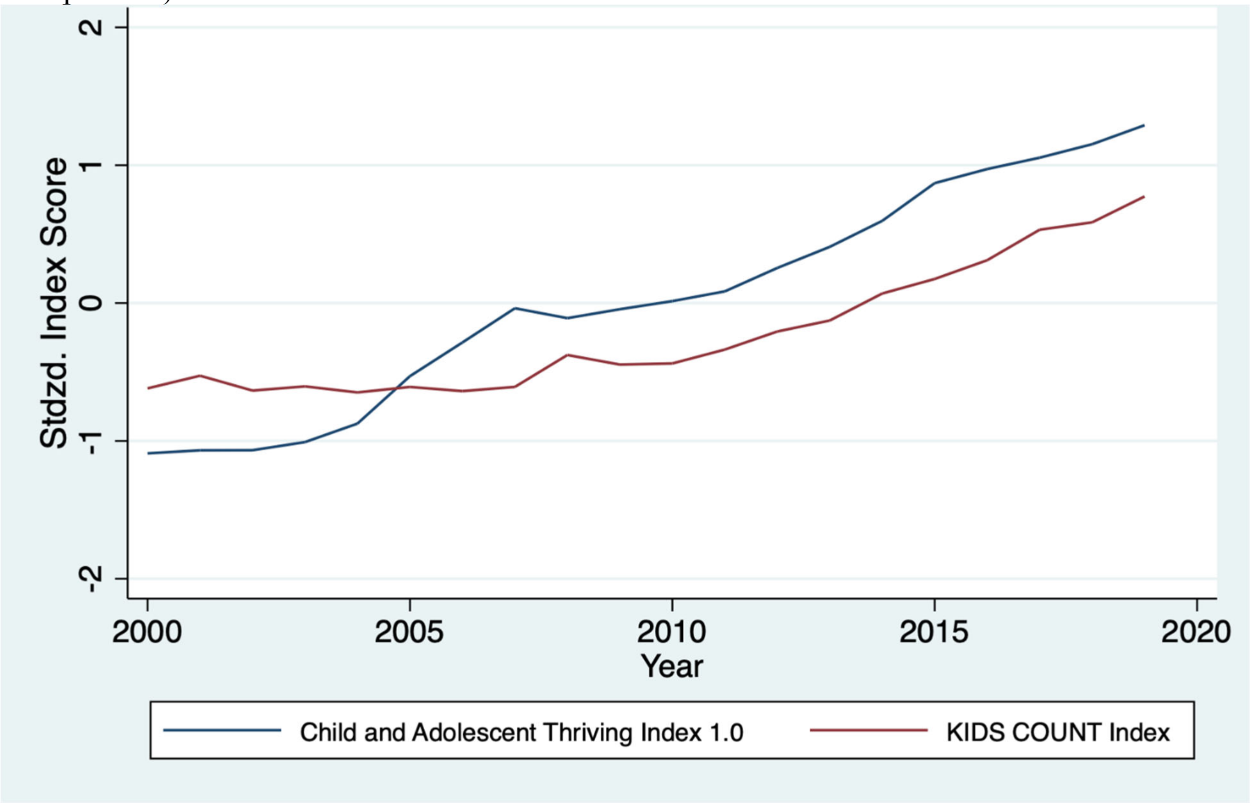

Notes: Both indices are standardized in this graph to facilitate comparability.

© 2022 Anderson NW et al. *JAMA Network Open.*

eFigure 4. Robustness of Data Standardization Methods Comparing With Version Where Components Are Normalized

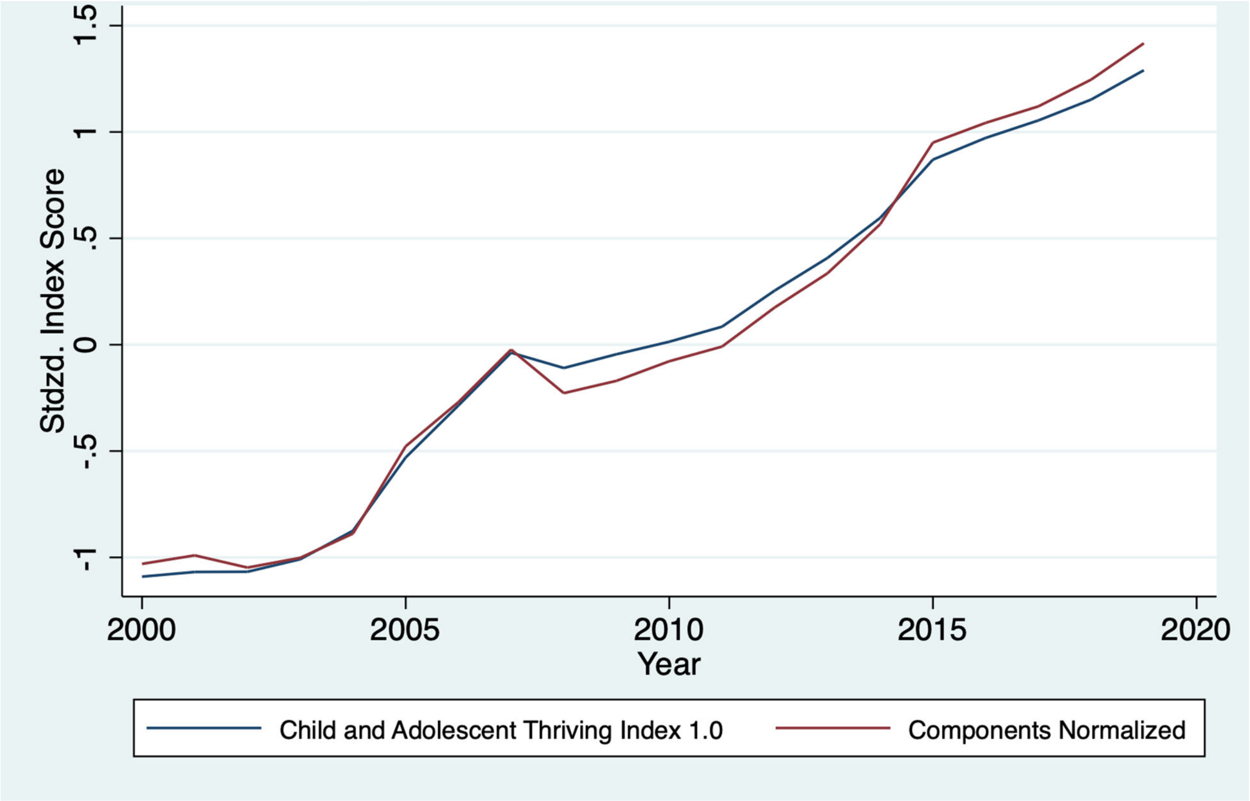

Notes: Both indices are standardized in this graph to facilitate comparability.

© 2022 Anderson NW et al. *JAMA Network Open.*

eFigure 5. Robustness of Aggregation Methods Comparing With Substitutability Adjustment

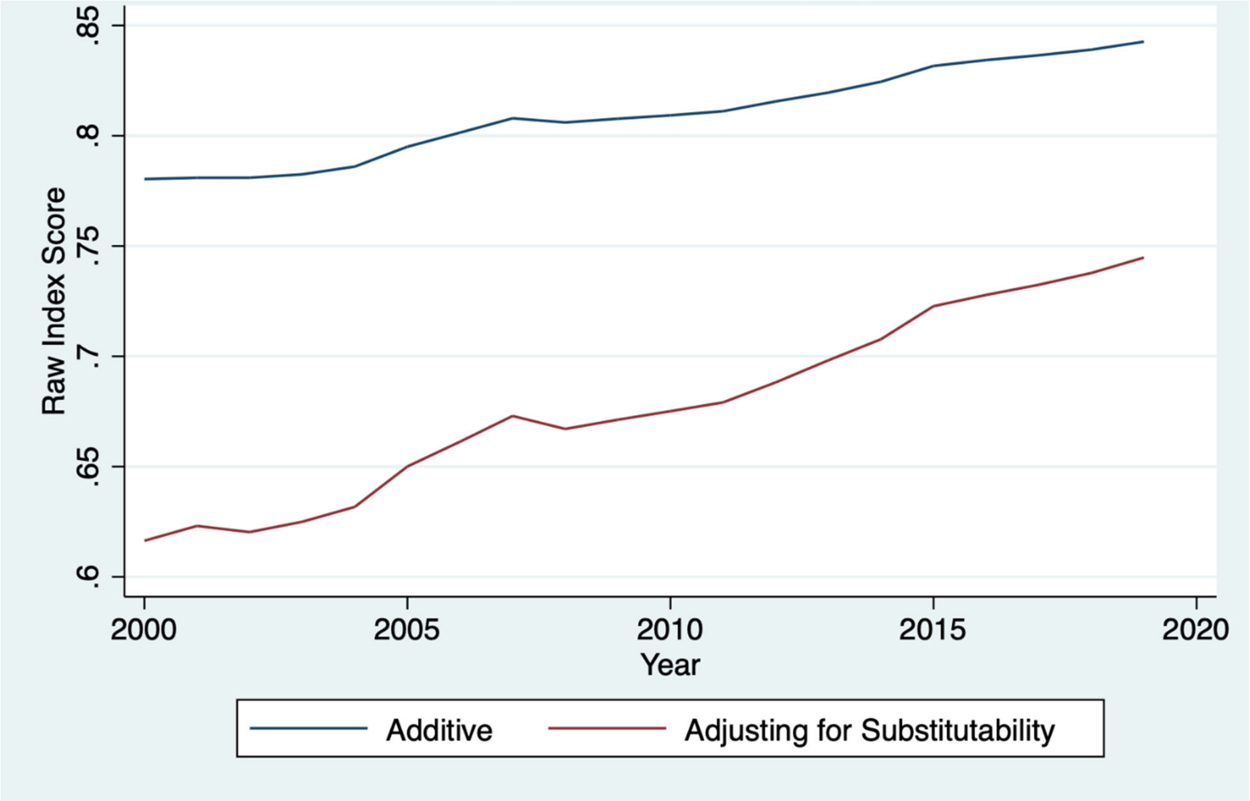

Notes: Index adjusting for substitutability is systematically lower because the index heavily penalizes instances in which one component is lower than the others. This also helps explain its greater relative improvement over time, since it is typically easier to improve outcomes with lower baseline values.

© 2022 Anderson NW et al. *JAMA Network Open.*

eFigure 6. 90% Credible Intervals of State Rankings for Select Years With Various Forms of Uncertainty

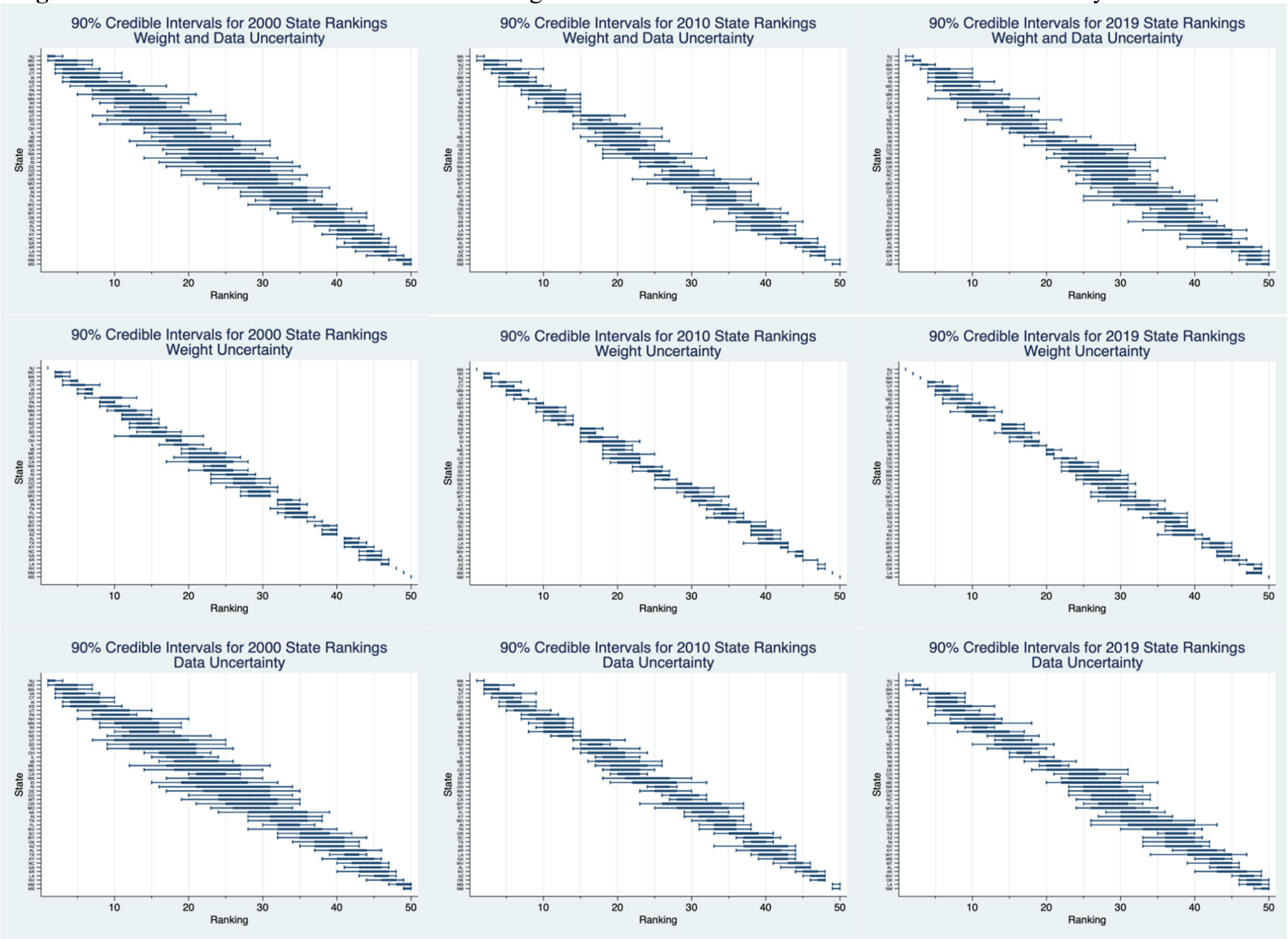

Notes: Credible intervals constructed from 10,000 Monte Carlo simulations.

© 2022 Anderson NW et al. *JAMA Network Open.*

eTable 10. Probability of Being Within 5 Ranks of Calculated Ranking by Source of Uncertainty, 2000-2019

	Weight and Data Uncertainty	Uncertainty related to Weights Only	Uncertainty related to Data Quality Only
Total	86.7%	98.7%	89.4%
Years			
2000-2004	79.8%	97.8%	82.6%
2005-2010	89.3%	99.2%	91.8%
2010-2014	90.2%	99.0%	92.8%
2015-2019	87.7%	98.7%	90.4%
Rank (Best to Worst)			
1-10	94.7%	99.7%	96.4%
11-20	85.7%	98.4%	88.7%
21-30	76.1%	96.8%	80.0%
31-40	82.6%	98.7%	85.9%
41-50	94.6%	99.8%	96.0%
Size (Largest to Smallest)			
1-10	92.6%	98.5%	95.8%
11-20	91.0%	99.4%	93.1%
21-30	89.1%	98.8%	91.9%
31-40	87.5%	98.7%	90.0%
41-50	73.5%	97.8%	76.3%

Notes: Estimates based on 10,000 Monte Carlo simulations.

© 2022 Anderson NW et al. *JAMA Network Open.*

eTable 11. Margin of Error Analysis: Number of Ranks Above or Below Calculated Rank, For Which There Is a 90% Probability of the True Rank, by Source of Uncertainty, 2000-2019

	Weight and Data Uncertainty	Uncertainty related to Weights Only	Uncertainty related to Data Quality Only
Total	± 6	± 3	± 6
Years			
2000-2004	± 8	± 3	± 7
2005-2010	± 6	± 3	± 5
2010-2014	± 5	± 3	± 5
2015-2019	± 6	± 3	± 5
Rank (Best to Worst)			
1-10	± 4	± 2	± 4
11-20	± 6	± 3	± 6
21-30	± 8	± 4	± 7
31-40	± 7	± 3	± 6
41-50	± 4	± 2	± 4
Size (Largest to Smallest)			
1-10	± 5	± 3	± 4
11-20	± 5	± 3	± 5
21-30	± 6	± 3	± 5
31-40	± 6	± 3	± 6
41-50	± 9	± 3	± 8

Notes: Estimates based on 10,000 Monte Carlo simulations.

© 2022 Anderson NW et al. *JAMA Network Open.*

eFigure 7. Robustness of Selection of Indicators Comparison With Contextual Variable Index

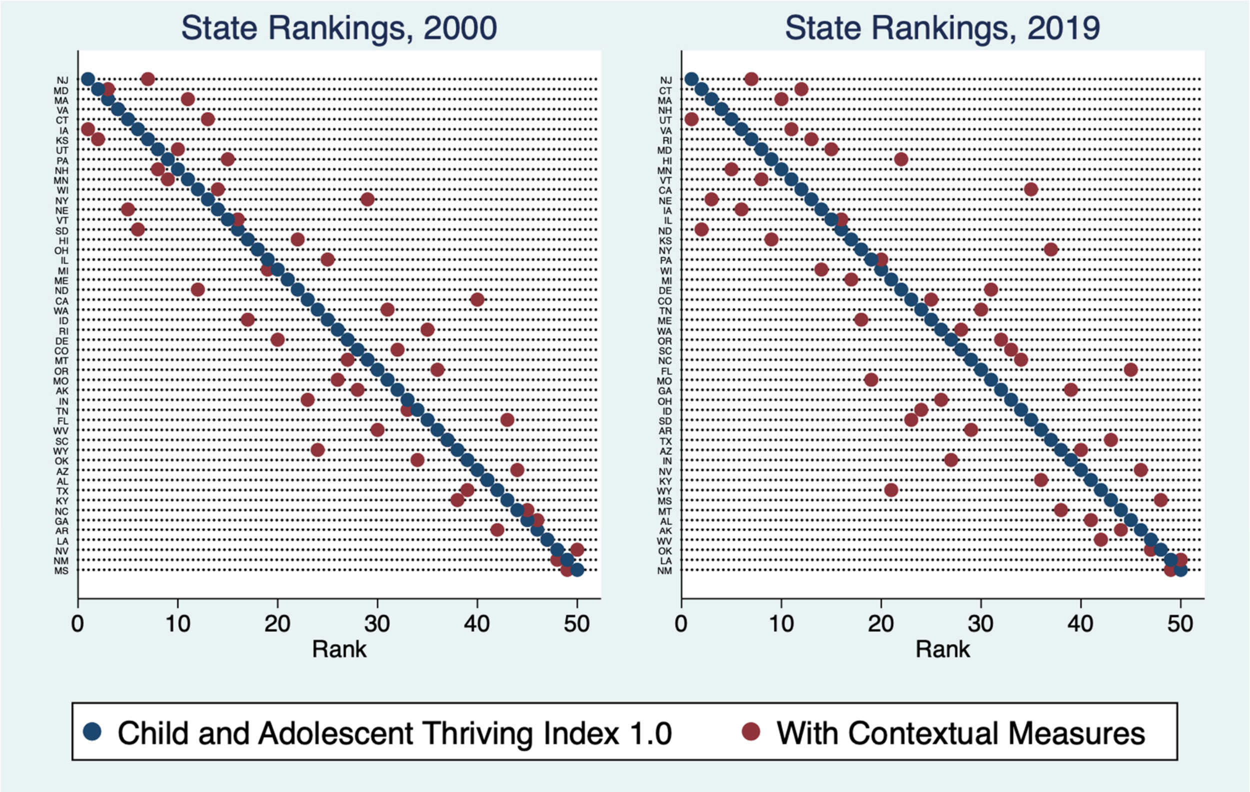

© 2022 Anderson NW et al. *JAMA Network Open.*

eFigure 8. Robustness of Selection of Indicators Comparison With Alternative Model Selection Procedure

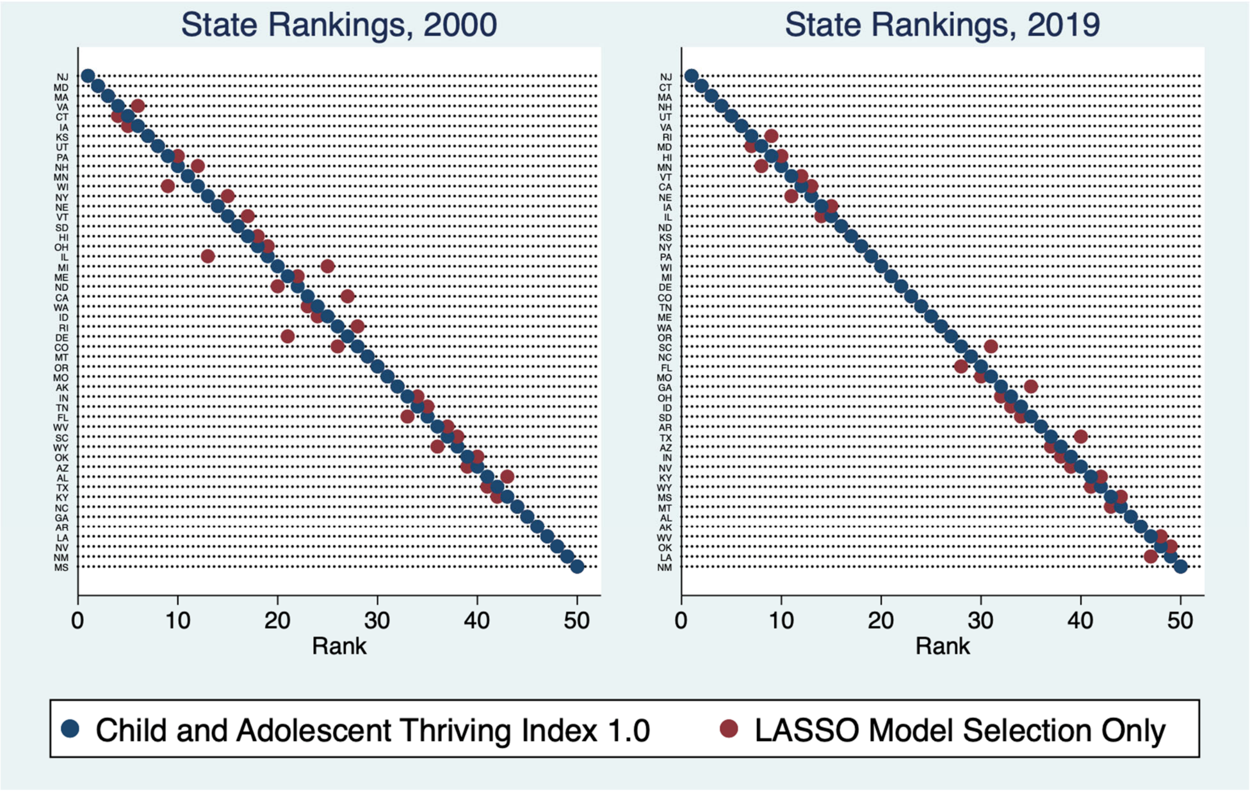

© 2022 Anderson NW et al. *JAMA Network Open.*

eFigure 9. Robustness of Weighting Scheme Comparing to KIDS COUNT Ranks (Equally Weighted Components)

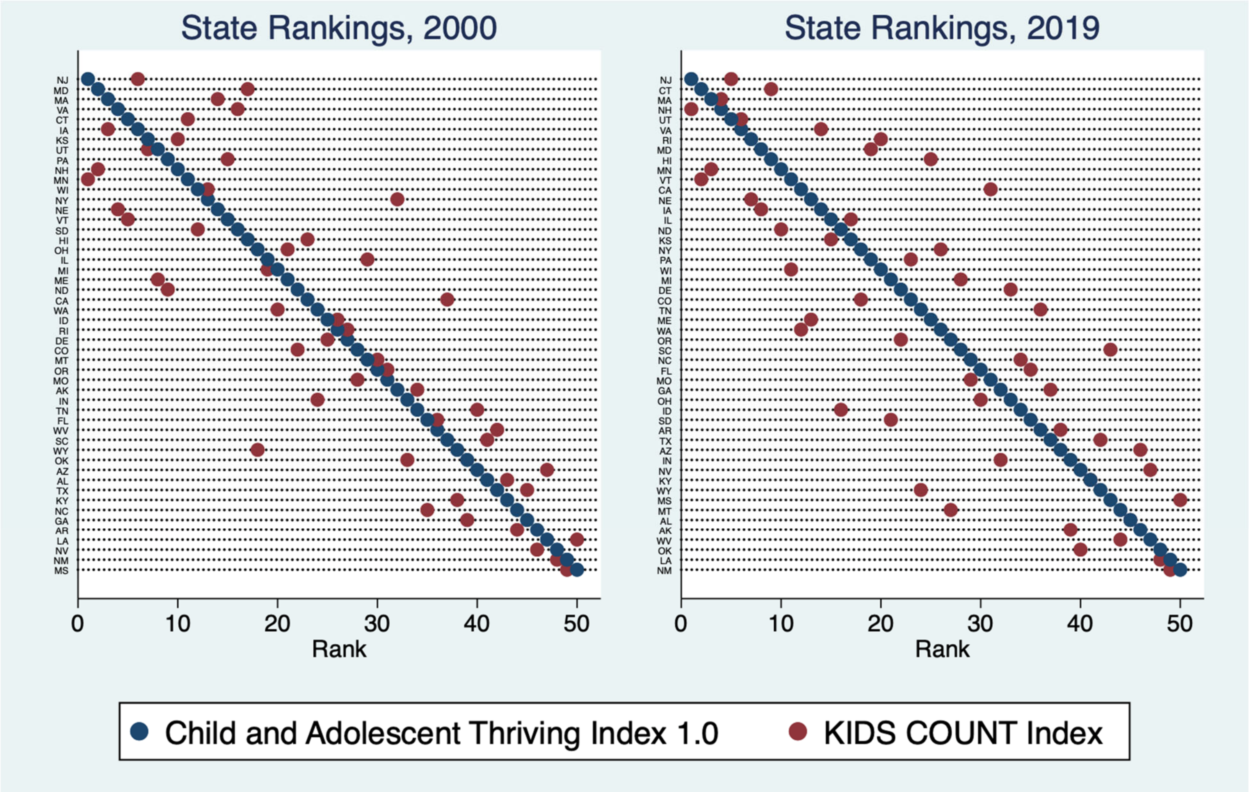

© 2022 Anderson NW et al. *JAMA Network Open.*

eFigure 10. Robustness of Data Standardization Methods Comparing With Version Where Components Are Normalized

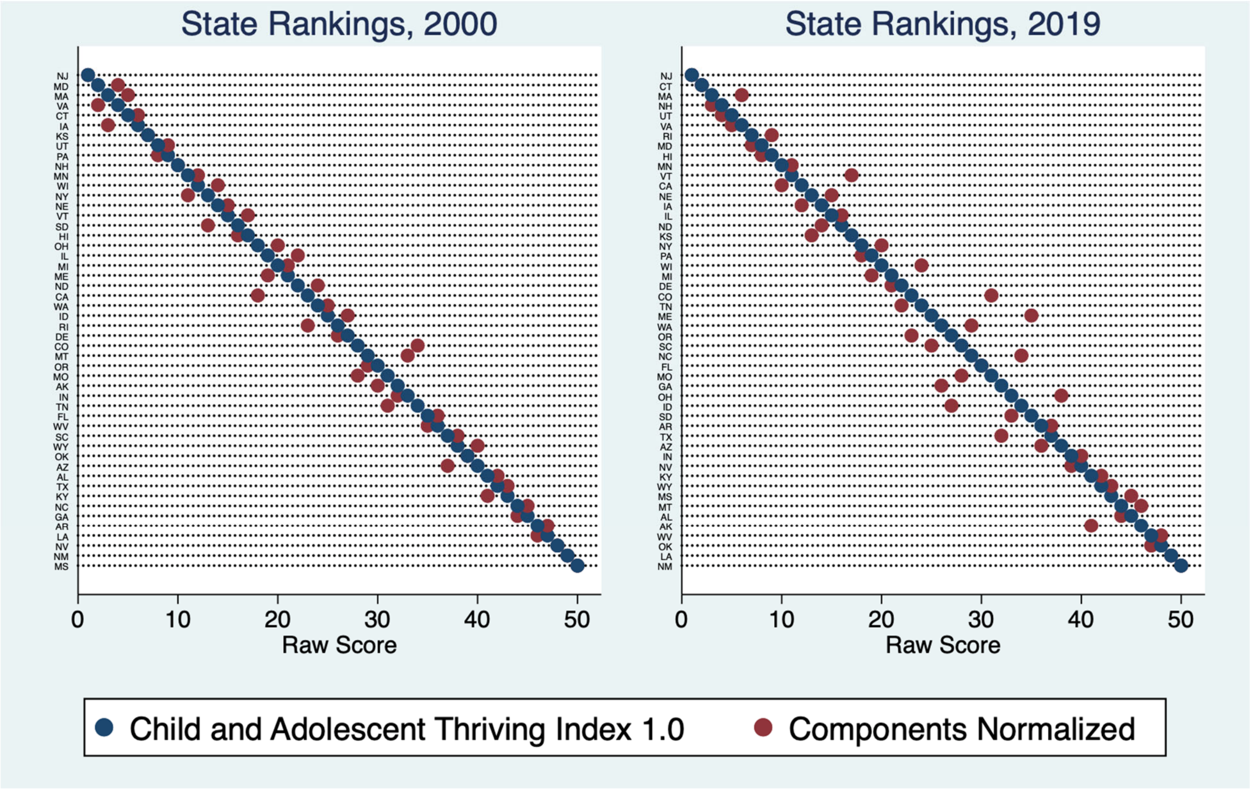

© 2022 Anderson NW et al. *JAMA Network Open.*

eFigure 11. Robustness of Aggregation Methods Comparing With Substitutability Adjustment

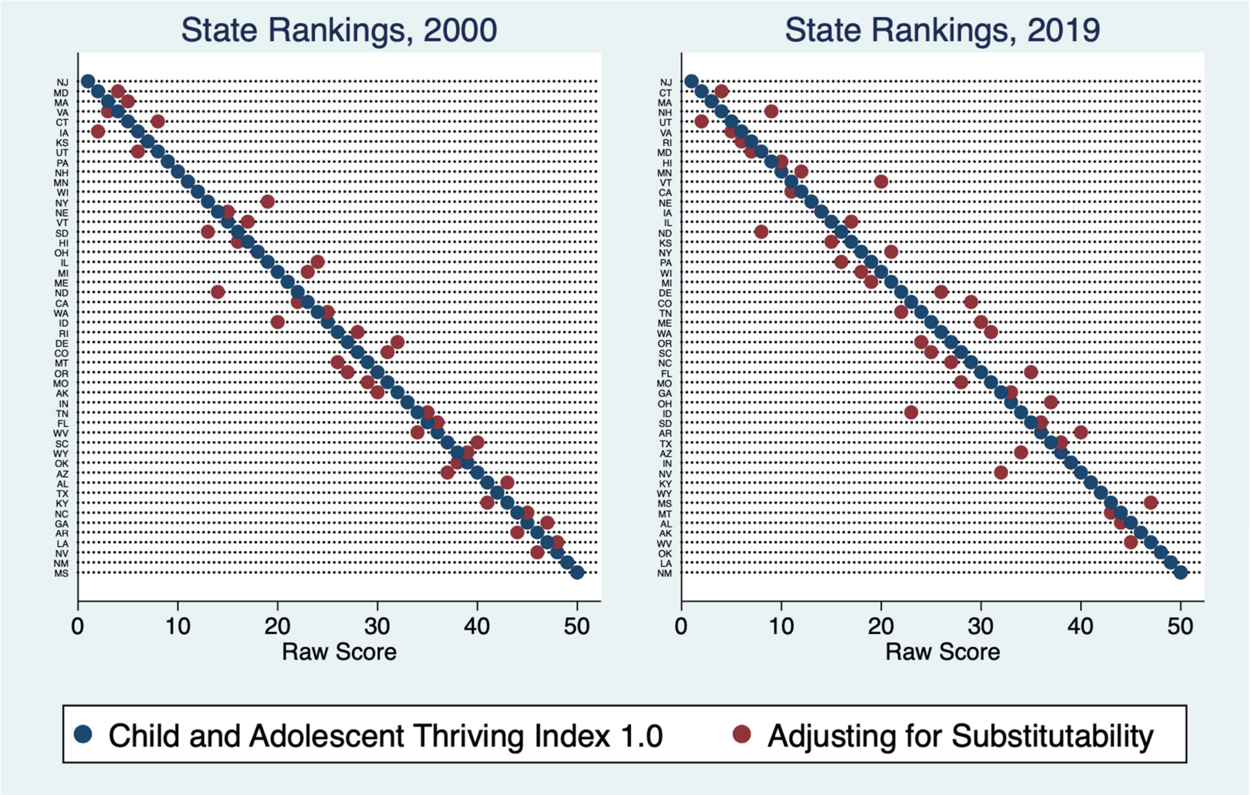

© 2022 Anderson NW et al. *JAMA Network Open.*

eFigure 12. Convergence in State-Level Child and Adolescent Thriving Index 1.0, 2000-2019

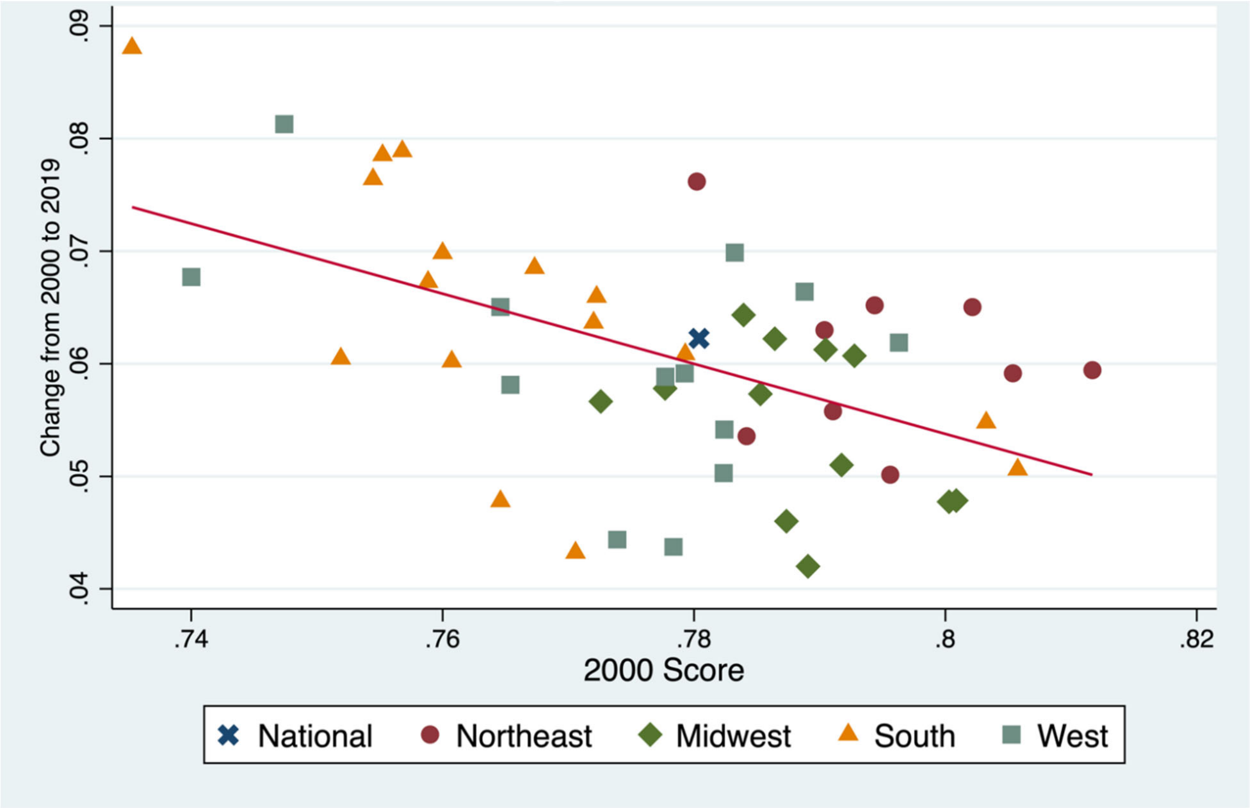

Notes: Downward slope of red line indicates overall convergence in state-wellbeing scores on average over time.

© 2022 Anderson NW et al. *JAMA Network Open.*

eFigure 13. State-Level Child and Adolescent Thriving Index 1.0, Select Years

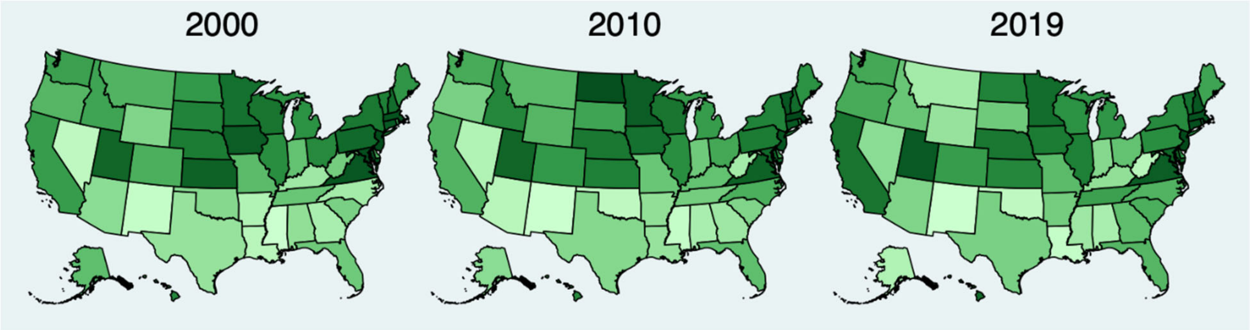

Notes: Darker shades correspond to higher rankings in child and adolescent well-being.

© 2022 Anderson NW et al. *JAMA Network Open.*

eTable 12. Rank Differences Across Annie E. Casey KIDS COUNT Index (AEC) and Child and Adolescent Thriving Index 1.0 (CATI-1.0)

State	2000			2010			2019		
	AEC	CATI-1.0	Diff.	AEC	CATI-1.0	Diff.	AEC	CATI-1.0	Diff.
Alabama	43	41	2	46	45	1	45	45	0
Alaska	34	32	2	26	40	-14	39	46	-7
Arizona	47	40	7	45	47	-2	46	38	8
Arkansas	44	46	-2	44	41	3	38	36	2
California	37	23	14	35	29	6	31	12	19
Colorado	22	28	-6	24	22	2	18	23	-5
Connecticut	11	5	6	9	5	4	9	2	7
Delaware	25	27	-2	25	24	1	33	22	11
Florida	36	35	1	42	32	10	35	30	5
Georgia	39	45	-6	43	43	0	37	32	5
Hawaii	23	17	6	19	18	1	25	9	16
Idaho	26	25	1	21	17	4	16	34	-18
Illinois	29	19	10	22	19	3	17	15	2
Indiana	24	33	-9	32	35	-3	32	39	-7
Iowa	3	6	-3	5	11	-6	8	14	-6
Kansas	10	7	3	17	15	2	15	17	-2
Kentucky	38	43	-5	39	33	6	41	41	0
Louisiana	50	47	3	47	42	5	48	49	-1
Maine	8	21	-13	11	20	-9	13	25	-12
Maryland	17	2	15	13	9	4	19	8	11
Massachusetts	14	3	11	6	1	5	4	3	1
Michigan	19	20	-1	33	23	10	28	21	7
Minnesota	1	11	-10	4	6	-2	3	10	-7
Mississippi	49	50	-1	50	49	1	50	43	7
Missouri	28	31	-3	27	34	-7	29	31	-2
Montana	30	29	1	28	31	-3	27	44	-17
Nebraska	4	14	-10	8	13	-5	7	13	-6
Nevada	46	48	-2	48	46	2	47	40	7
New Hampshire	2	10	-8	2	10	-8	1	4	-3
New Jersey	6	1	5	7	3	4	5	1	4
New Mexico	48	49	-1	49	50	-1	49	50	-1
New York	32	13	19	30	16	14	26	18	8
North Carolina	35	44	-9	34	28	6	34	29	5
North Dakota	9	22	-13	3	2	1	10	16	-6
Ohio	21	18	3	29	26	3	30	33	-3
Oklahoma	33	39	-6	36	48	-12	40	48	-8
Oregon	31	30	1	31	37	-6	22	27	-5
Pennsylvania	15	9	6	20	14	6	23	19	4
Rhode Island	27	26	1	23	21	2	20	7	13
South Carolina	41	37	4	40	38	2	43	28	15
South Dakota	12	16	-4	16	25	-9	21	35	-14
Tennessee	40	34	6	37	36	1	36	24	12
Texas	45	42	3	41	39	2	42	37	5
Utah	7	8	-1	10	8	2	6	5	1
Vermont	5	15	-10	1	4	-3	2	11	-9
Virginia	16	4	12	14	7	7	14	6	8
Washington	20	24	-4	18	27	-9	12	26	-14
West Virginia	42	36	6	38	44	-6	44	47	-3
Wisconsin	13	12	1	12	12	0	11	20	-9
Wyoming	18	38	-20	15	30	-15	24	42	-18

© 2022 Anderson NW et al. *JAMA Network Open.*

eFigure 14. Disparities with White Children, National Child and Adolescent Thriving Index 1.0, 2000-2019

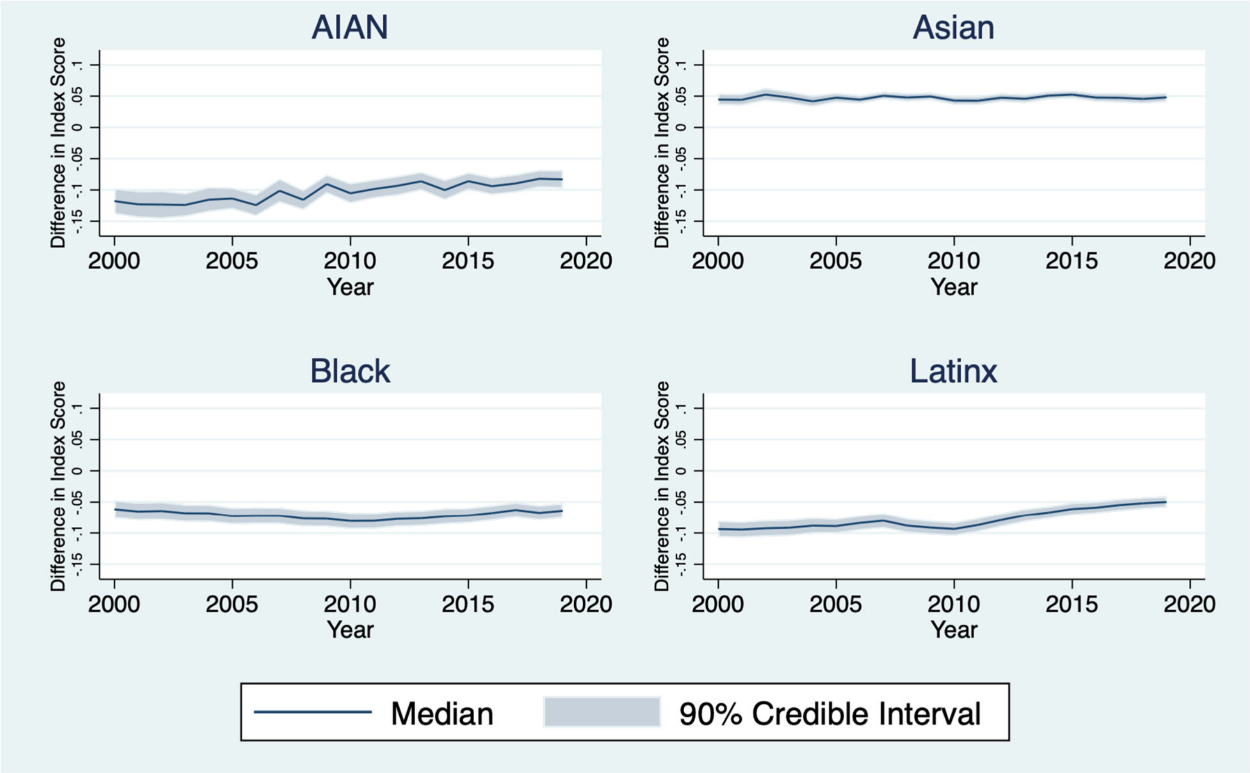

Notes: Credible interval is based on 10,000 Monte Carlo simulations for both weights' values and data variability.

© 2022 Anderson NW et al. *JAMA Network Open.*

eTable 13. Pairwise Correlation Analysis of Changes in State-Level Index Component Values from 2000-2019

	Food Secure	Preschool	High School	Math	Reading	Non-Obese	Health Status	LBW	Cigarettes	Marijuana	Arrests
Food Secure	–										
Preschool	0.0549	–									
High School	-0.0882	0.1017	–								
Math	0.2112	-0.2120	0.0454	–							
Reading	0.3980	-0.3742	0.0879	0.5693	–						
Non-Obese	-0.0393	0.1617	0.0250	0.0172	-0.1498	–					
Health Status	0.0636	-0.0115	-0.2243	0.1086	-0.1969	0.3499	–				
LBW	0.0371	0.1172	-0.3197	0.0836	0.0542	-0.0130	-0.0677	–			
Cigarettes	-0.5180	-0.1267	0.0530	-0.2624	-0.2934	-0.1246	0.0148	-0.0328	–		
Marijuana	-0.3770	-0.2439	-0.2091	0.0757	-0.0522	-0.0585	0.1469	-0.0124	0.4231	–	
Arrests	-0.0591	0.0867	-0.0805	0.0027	-0.0081	0.2286	-0.0722	0.1225	0.0767	-0.1909	–

Notes: Pairwise correlation of state-level changes in component values from 2000-2019. Components are unadjusted, meaning we do not multiply the change by the index weight.

© 2022 Anderson NW et al. *JAMA Network Open.*

eFigure 15. Decomposing Change in National Child and Adolescent Thriving Index 1.0 by Race/Ethnicity

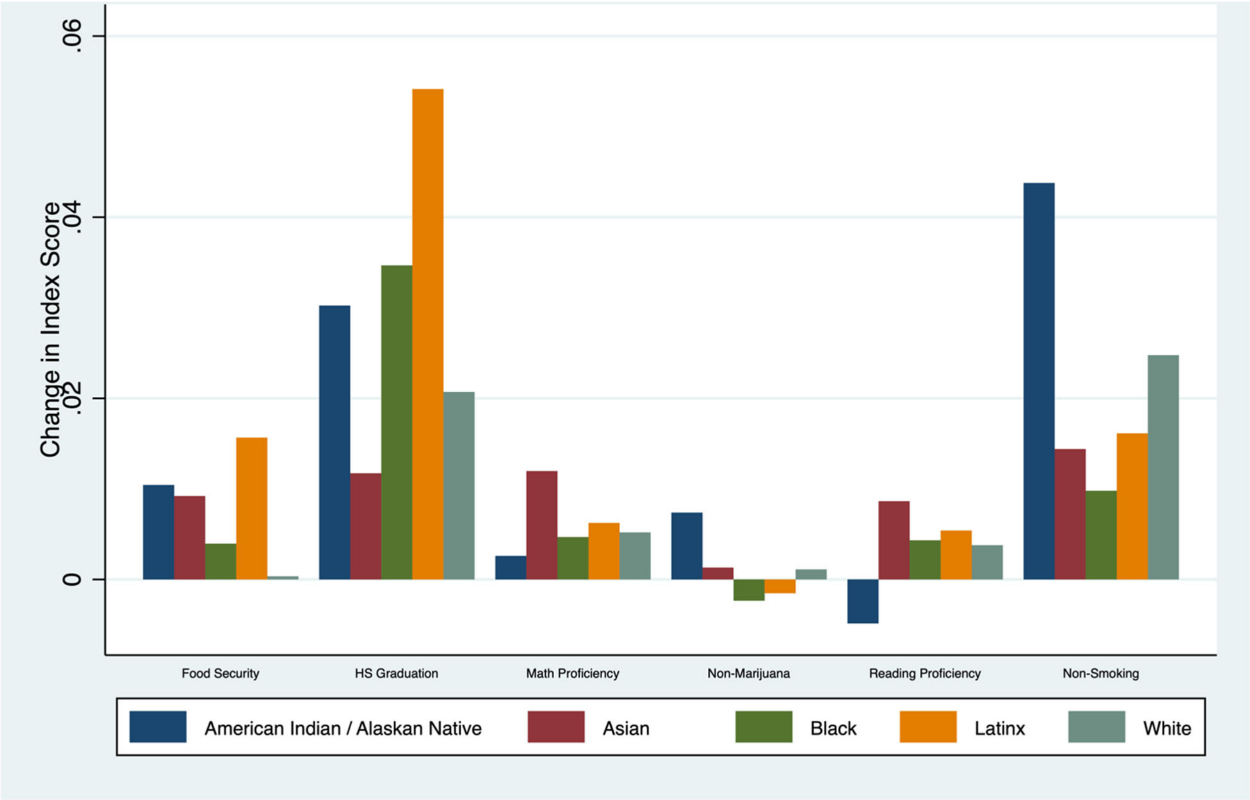

Notes: Estimates here are the calculated as the change in component prevalence from 2000 to 2019, multiplied by the respective component weight. Select outcomes (general health, non-low birthweight, non-obesity, and preschool) are omitted since the change in index score is relatively small.

© 2022 Anderson NW et al. *JAMA Network Open.*

eFigure 16. Comparison When Using ACGR Measure

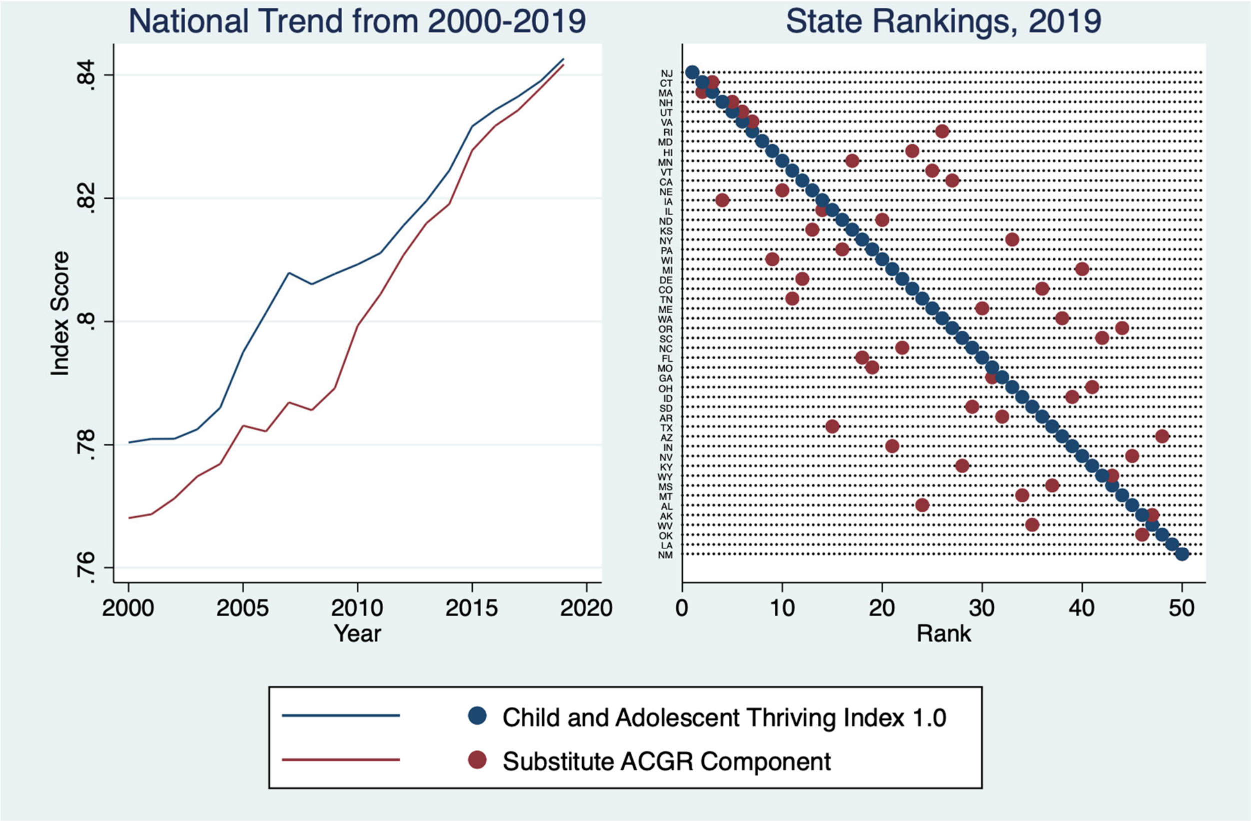

Notes: ACGR measure substitutes the Adjusted Cohort Graduation Rate for the high school completion measure used in the standard Child and Adolescent Thriving Index 1.0 (percent of persons 18-21 who remained in the same state as prior year that have a high school degree or more).

© 2022 Anderson NW et al. *JAMA Network Open.*

eReferences

1. Nardo M, Saisana M, Saltelli A, Tarantola S. *Tools for Composite Indicators Building.* Ispra, Italy: European Commission Joint Research Centre;2005.
2. O'Hare WP, Bramstedt NL. *Assessing The KIDS COUNT Composite Index.* 2003.
3. O'Hare WP. A Research Note on Statistical Methods Used to Create Indices of Child Well-Being. *Child Indicators Research.* 2015;8(2):279-298
4. Knippenberg R. The Dimensions of Ordinal Well-Being Indexes: Using Orthogonal Weighting with the Kids Count Index. 2017
5. Courtemanche C, Soneji S, Tchernis R. Modeling Area-Level Health Rankings. *Health services research.* 2015;50(5):1413-1431
6. Anderson NW, Markowitz AJ, Eisenberg D, Halfon N, Moore KA, Zimmerman FJ. The Child and Adolescent Thriving Index 1.0: Developing a Measure of the Outcome Indicators of Well-Being for Population Health Assessment. *Child Indicators Research.* 2022
7. Decancq K, Lugo MA. Weights in Multidimensional Indices of Wellbeing: An Overview. *Econometric Reviews.* 2013;32(1):7-34
8. Biggeri M, Ferrone L. Measuring Child Multidimensional Deprivation: A Sustainability Perspective. *Sustainability.* 2021;13(7):3922

© 2022 Anderson NW et al. *JAMA Network Open.*
